# Employment status and occupation are associated with liver cirrhosis: A nationwide Swedish case–control study

**DOI:** 10.1111/joim.70166

**Published:** 2026-10-07

**Authors:** Marie Zalesiak, Juan Vaz, Eralda Asllanaj, Martin Henriksson, Hannes Hagström, Ying Shang

**Affiliations:** ^1^ Department of Medicine, Huddinge Karolinska Institutet Stockholm Sweden; ^2^ Boehringer Ingelheim Pharma GmbH & Co. KG Ingelheim Germany; ^3^ School of Public Health and Community Medicine, Institute of Medicine, Sahlgrenska Academy University of Gothenburg Gothenburg Sweden; ^4^ Department of Internal Medicine Halland Hospital Halmstad Halmstad Sweden; ^5^ Boehringer Ingelheim B.V. Amsterdam the Netherlands; ^6^ Centre for Medical Technology Assessment, Department of Medical, Health and Caring Sciences Linköping University Linköping Sweden; ^7^ Unit of Hepatology, Department of Upper GI Diseases Karolinska University Hospital Stockholm Sweden

**Keywords:** case–control, case‐finding, cirrhosis, early detection, labor market, occupation

## Abstract

**Background:**

Liver cirrhosis is often diagnosed in working‐age adults and carries a poor prognosis. A better understanding of variation by employment status and occupation may support earlier and more targeted diagnostic evaluation.

**Objectives:**

To assess the odds of liver cirrhosis across labor‐market status and occupations in working‐age adults and whether these patterns differed by etiology.

**Methods:**

In this nationwide case–control study, individuals aged 35–65 years with cirrhosis during 2002–2021 were identified from Swedish national registries and matched to up to four controls on age, sex, and municipality. Labor‐market status in the year before diagnosis was classified as employed, unemployed, disability/activity benefit, long‐term sick leave, or other. Employed individuals were classified into occupations. Conditional logistic regression was used to estimate odds ratios and 95% confidence intervals, with physical and engineering professionals as the reference group.

**Results:**

We included 23,033 cases and 92,106 controls. Cases were less often employed than controls (25.7% vs. 66.2%) and more often belonged to labor‐market strata associated with a lower socioeconomic status. In analyses, including employed and unemployed individuals (7485 cases; 63,747 controls), unemployment was associated with the highest odds of cirrhosis. Among employed individuals, food preparation workers and refuse and recycling workers had the highest odds. Stratified by etiologies, associations were strongest for alcohol‐related cirrhosis. Patterns across sex strata were similar, with generally larger effect sizes in men.

**Conclusion:**

Among working‐age adults, unemployment and selected occupations were strongly associated with incident cirrhosis. Routinely collected employment information may help identify groups who could benefit from earlier liver evaluation.

## Graphical abstract

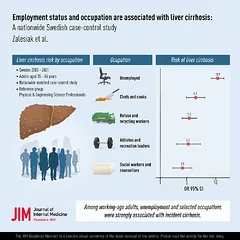

**Abbreviations**
ALD: alcohol‐related liver disease
CDR: cause of death registry
CI: confidence interval
DELIVER: DEcoding the Epidemiology of Liver Disease in Sweden
FDR: false discovery rate
HBV: hepatitis B virus
HCV: hepatitis C virus
ICD‐10: International Classification of Disease, 10th Revision
ISCO‐08: International Standard Classification of Occupations 2008
LISA: longitudinal integration database for health insurance and labor market studies
MASLD: metabolic dysfunction–associated steatotic liver disease
NACE: Statistical Classification of Economic Activities in the European Community
NPR: National Patient Registry
OR: odds ratio
SNI: Swedish Standard Industrial Classification
SSYK: Swedish Standard Classification of Occupations

## Introduction

Liver cirrhosis is a leading cause of premature mortality worldwide, and its incidence continues to rise in many high‐income countries, including Sweden [1, 2]. A substantial proportion of patients are still first recognized only after developing liver cirrhosis and hepatocellular carcinoma, when prognosis is already poor and therapeutic options are limited [3]. Improving outcomes therefore depends on earlier identification of individuals who are at risk of progressing to cirrhosis before it becomes clinically apparent. Current guidelines support the use of noninvasive tests to assess liver fibrosis in people at increased risk, yet implementation in routine care remains modest at best [4, 5, 6]. One of the practical challenges is therefore how to identify patients who need further liver evaluation outside traditional hepatology settings.

This is particularly relevant for working‐age adults, among whom chronic liver disease is the second leading cause of working years lost in Europe [4]. Previous studies have shown clear socioeconomic gradients in cirrhosis and other adverse liver outcomes, with a higher burden in more disadvantaged groups [2, 7, 8]. Most of this literature has focused on broad indicators such as income, education, or area‐level deprivation, but this information is not always available in clinical encounters. In contrast, employment status can be easily ascertained during routine history taking. It may serve as a simple and pragmatic marker of vulnerability, whereas detailed occupation information may provide additional layer of long‐term work‐related exposures to liver disease risk and might complement conventional assessments [5, 9].

At the same time, the clinical relevance of labor‐market position and occupation for cirrhosis case‐finding remains poorly understood. Previous occupational studies have largely focused on specific toxic exposures or selected liver disease etiologies, with fewer studies examining cirrhosis as a clinical endpoint [10, 11, 12]. We therefore used nationwide Swedish register data to describe the distribution of incident cirrhosis across labor‐market categories and to estimate whether unemployment and, among employed, certain occupations were associated with higher odds for liver cirrhosis overall and by etiology. By identifying labor‐market and occupational groups in which cirrhosis is concentrated, this study aims to provide an evidence‐generating basis for future evaluation of occupation‐informed case‐finding strategies.

## Materials and methods

### Study design and data sources

We conducted a nationwide register‐based case–control study within the DEcoding the epidemiology of Liver Disease in Sweden (DELIVER) cohort, a liver disease‐specific registry cohort that links Swedish national health and population registries on the individual level using pseudonymized identification numbers [13]. Cirrhosis diagnoses and decompensation events were identified from the National Patient Registry (NPR) and the Cancer Registry, and deaths from the cause of death registry (CDR) [14]. Socioeconomic information was retrieved from the longitudinal integration database for health insurance and labor market studies (LISA), and demographic data and the municipality of residence were obtained from the Register of the Total Population [15, 16]. Additional registry details are provided in the Supporting Information.

### Study population

All individuals born between 1937 and 1986 who were residents in Sweden were eligible to capture established working careers (35–65 years old when enter the cohort). A minimal 1‐year look‐back period was applied to exclude individuals with prevalent cirrhosis. The study period began on January 1, 2002, and follow‐up continued until cirrhosis diagnosis, December 31, 2021, emigration, or death, whichever occurred first.

Incident cirrhosis cases were identified through the NPR and the CDR. The index date was defined as the earliest of (i) the first recorded ICD‐10 diagnosis of cirrhosis in the NPR; (ii) the first recorded decompensation event consistent with cirrhosis in the absence of a prior cirrhosis code; or (iii) the date of death when cirrhosis was listed as an underlying or contributing cause in the CDR and no prior cirrhosis diagnosis was recorded in the NPR. ICD‐10 definitions for cirrhosis and decompensation events are provided in Table S1. Up to four controls per case were selected using incidence‐density sampling and matched on birth year, sex, municipality of residence, and calendar year of the case index date. Controls were required to be alive and free of liver disease on the corresponding index date (Fig. 1).

**Fig. 1:**
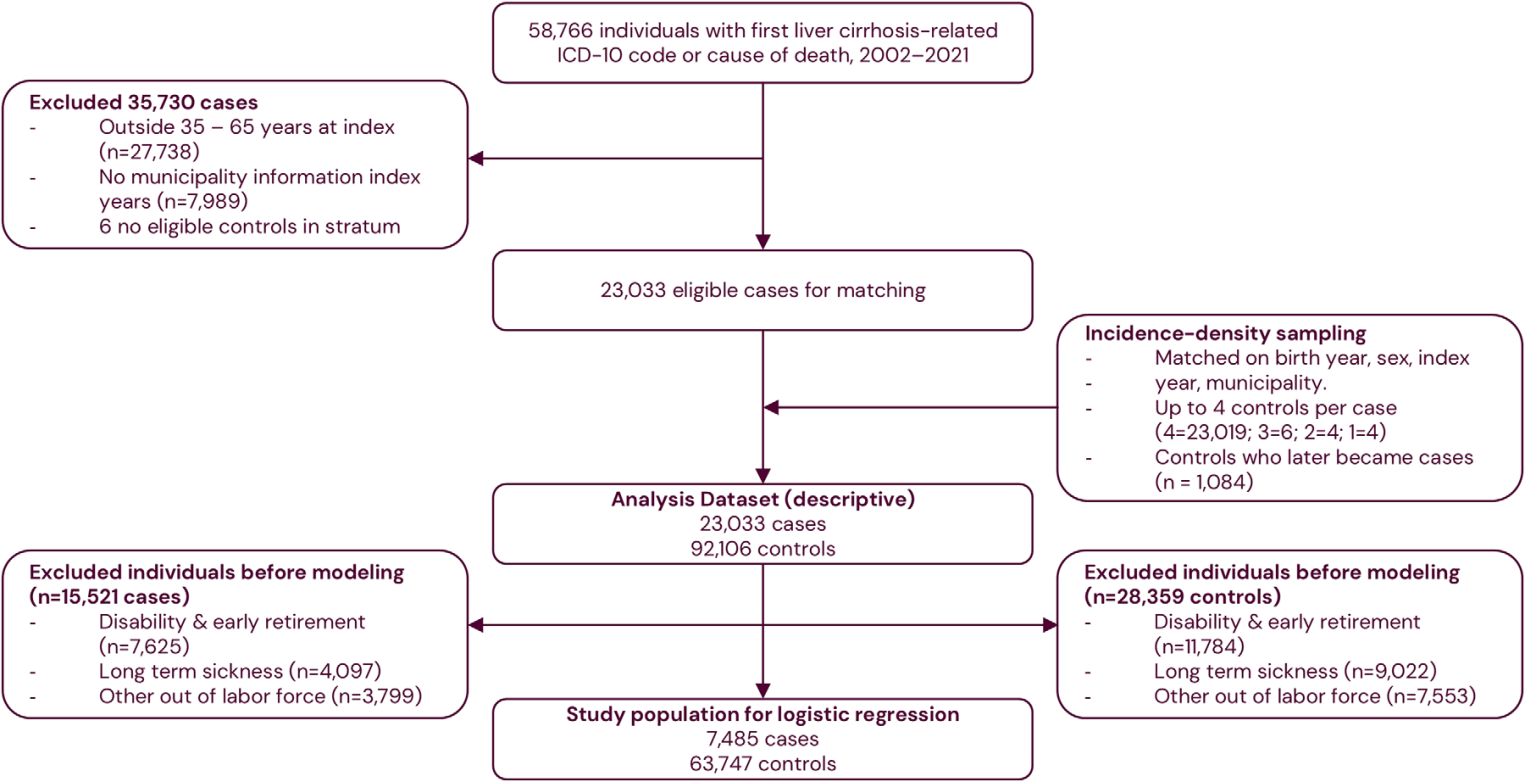
Study population, exclusions, and construction of the case–control cohort. Individuals from the DELIVER cohort born 1937–1986 were screened for cirrhosis‐related ICD‐10 diagnosis or cause of death during 2002–2021. After applying event‐level exclusions (washout and decompensation/ascites rules), 58,766 individuals remained. Excluding age and data‐availability criteria, 23,033 incident cirrhosis cases remained eligible for incidence‐density sampling. Up to four controls per case were matched on birth year, sex, calendar year of index, and municipality. Individuals with disability or early retirement, long‐term sickness, or classified as other were excluded before modeling, resulting in a final study population of 7485 cases and 63,747 controls for logistic regression analyses.

### Case ascertainment and etiologic classification

Our primary outcome was all‐cause cirrhosis. The secondary outcome was cirrhosis due to different etiologies. For each incident cirrhosis, we assigned an underlying etiology using ICD‐10 codes from the NPR or, when applicable, from the CDR. Etiological codes were considered within ±90 days from the index date to capture diagnostic work‐up at presentation. For individuals without a documented code for a specific underlying etiology, the presence of cardiometabolic risk factors was used to define metabolic dysfunction–associated steatotic liver disease (MASLD) [17]. When multiple etiologies were present, assignment followed a hierarchy: viral hepatitis B or viral hepatitis C; ALD; alcohol‐related liver disease (primary biliary cholangitis or autoimmune hepatitis or primary sclerosing cholangitis; toxic or drug‐induced; MASLD; rare genetic; and cryptogenic causes). ICD codes used for definitions are provided in Table S1.

### Labor‐market status and occupation

Labor‐market information for 2002–2021 was obtained from LISA for the calendar year preceding the index date. To capture each individual's main labor‐market position before diagnosis, status was assigned hierarchically into five mutually exclusive categories: disability/activity benefit, long‐term sick leave, unemployed, employed, and other. Individuals were first classified as receiving disability or activity benefits if any disability pension, activity compensation, or related early‐retirement benefit was recorded. Among the remainder, those with any recorded sickness benefit were classified as on long‐term sick leave. Among those not classified in either of these categories, unemployment was defined using the LISA unemployment code (ALKod) in combination with employment status (SyssStat) at the person‐year level. The remaining individuals recorded as gainfully employed were classified as employed. All remaining individuals were classified as other, including students, homemakers, individuals on parental or other family‐related leave, participants in labor‐market programs not classified as unemployed, and others outside the labor force. This hierarchical approach reduced overlap between categories and yielded a strict definition of current employment. To account for the 2003 reform of the Swedish sickness‐benefit system, long‐term sick leave indicators before 2003 were reclassified as disability or activity benefit [16].

Among employed individuals, an occupational classification was defined using the Swedish Standard Classification of Occupations (SSYK), which is based on the International Standard Classification of Occupations 2008 (ISCO‐08) [18]. Occupational classifications were harmonized across coding systems to SSYK‐2012 at the three‐digit level, with prespecified aggregation of sparse or closely related groups. Those with a valid three‐digit occupational code were assigned to occupational groups, whereas those without a recorded occupational code were retained as a separate category. For descriptive presentation, occupational groups were additionally mapped to broader industry domains according to the European statistical classification of economic activities (NACE) implemented in Sweden as SNI 2007 classifications [19, 20]. Full coding rules, harmonization procedures, and occupational mappings are provided in Supporting Information (pp. 1–2) and Tables S2–S4, Fig. S1.

Employment history before cirrhosis diagnosis was summarized using two indicators: The proportion of observed years spent unemployed, as a marker of labor market attachment, and the rate of change between occupational codes across observed years in employment, as a marker of occupational stability.

### Covariates

The sociodemographic covariates were obtained from the calendar year preceding the index date unless stated otherwise. Education was classified into three levels according to the Swedish National Education Nomenclature (<10 years; 10–12 years; >12 years) [16]. Disposable income per household per consumption unit was taken from LISA (posttax, post‐transfer annual income) and categorized based on quintiles after log transformation to address skewness. Country of birth was coded as Nordic versus non‐Nordic.

### Statistical analyses

Baseline characteristics were summarized using means (SDs) for continuous variables and frequencies (%) for categorical variables. Conditional logistic regression was used to estimate odds ratios (ORs) and 95% confidence intervals (CIs) for incident cirrhosis, accounting for the matching pairs. The primary exposure analyses focused on two complementary exposure dimensions: Unemployment and occupational groups among individuals who were employed. Physical and engineering science professionals were chosen as the reference group because of the observed low event rate and homogeneity. Physical and engineering science professionals included occupations such as physicists, chemists, mathematicians, statisticians, and civil engineers. We reported ORs from both the crude model and the model for household income and education, and then additionally for longitudinal work‐history measures. As a sensitivity analysis, occupation was reassessed using information recorded 5 years before the index date. Secondary analyses were stratified by etiology, sex, and age group. To account for multiple comparisons across occupational groups, *p*‐values were adjusted using the Benjamini–Hochberg false discovery rate (FDR) procedure. Analyses were conducted in R version 4.4.2. During the preparation of this work the authors used Grammarly to support language editing. After using this tool, the authors reviewed and edited the content as needed and take full responsibility for the content of the published article.

## Results

### Baseline characteristics and labor‐market distribution

We analyzed 23,033 incident cirrhosis cases and 92,106 matched controls between 2002 and 2021. Mean age at index was 55 years in both groups, and 34.4% were women. Compared with controls, individuals with cirrhosis had lower educational attainment and lower disposable household income. Alcohol‐related liver disease was the most common etiology (32.7%), followed by viral hepatitis (28.4%) and MASLD (12.6%) (Table 1).

**Table 1:** Baseline characteristics.

Parameters	Cases	Controls
Included persons	23,033	92,106
Mean age at index (SD)	55 (7.3)	55 (7.3)
Sex		
Female	7914 (34.4%)	31,646 (34.4%)
Male	15,119 (65.6%)	60,460 (65.6%)
Region of birth		
Nordic	19,706 (85.6%)	79,758 (86.6%)
Non‐Nordic	3327 (14.4%)	12,348 (13.4%)
Socioeconomic status education		
<10 years	6674 (29.0%)	17,785 (19.3%)
10–12 years	11,607 (50.4%)	43,141 (46.8%)
>12 years	4274 (18.6%)	30,468 (33.1%)
Income		
Mean income (SD)	2008 (2567)	2803 (5930)
Disposable household income quintile		
Q1 (lowest)	8238 (35.8%)	14,783 (16.0%)
Q2	5606 (24.3%)	17,385 (18.9%)
Q3	3814 (16.6%)	19,146 (20.8%)
Q4	2874 (12.5%)	20,096 (21.8%)
Q5 (highest)	2354 (10.2%)	20,630 (22.4%)
No income history	147 (0.6%)	66 (0.1%)
Labor status		
Disability or activity benefit	7652 (33.2%)	11,784 (12.8%)
Long‐term sick leave	4097 (17.8%)	9022 (9.8%)
Unemployed	1571 (6.8%)	2765 (3.0%)
Other	3799 (16.5%)	7553 (8.2%)
Employed	5914 (25.7%)	60,982 (66.2%)
Employed with missing occupation (%)	382 (6.5%)	1196 (2.0%)
Mean total job changes (SD)	0.3 (0.8)	0.6 (1.1)
Mean observed years (SD)	10.3 (5.8)	10.6 (5.8)
Mean years unemployed (SD)	0.8 (1.7)	0.4 (1.2)
Etiology		
Viral hepatitis	6552 (28.4%)	–
ALD	7534 (32.7%)	–
MASLD	2899 (12.6%)	–
Autoimmune	1156 (5.0%)	–
Rare genetic	72 (0.3%)	–
Toxic	204 (0.9%)	–
Not identifiable	4616 (20.0%)	–

*Note*: Values are numbers (percentages) unless otherwise indicated. Socioeconomic indicators refer to the year before index. Labor market status is mutually exclusive and based on main income source. Etiologic subgroups are shown for cases only.

Abbreviation: MASLD, metabolic dysfunction–associated steatotic liver disease.

Labor‐market status differed markedly between cases and controls. Cases were less often employed in the pre‐index year than controls (25.7% vs. 66.2%). More than half of cases (51%) received sickness‐ or disability‐related payments, compared with 23% of controls. Among those who were employed, occupational coding was less complete in cases than in controls (6.5% vs. 2.0%).

### Unemployment and occupational associations

The primary analyses, focusing on employed and unemployed individuals, included 7485 cases and 63,747 controls. Compared with physical and engineering science professionals, unemployment was associated with the highest odds of cirrhosis (OR 10.9, 95% CI 8.3–14.1; FDR <0.01). Employed individuals without a specific recorded occupation also had substantially elevated odds (OR 6.7, 95% CI 5.0–9.0; FDR <0.001).

Among employed individuals with a recorded description of their occupation, the highest odds were concentrated in food‐related occupations, including butchers, bakers, and food processors (OR 4.9, 95% CI 2.1–11.6; FDR <0.001), chefs and cooks (OR 4.3, 95% CI 3.0–6.4; FDR <0.001), and head waiters, waiters, and bartenders (OR 3.7, 95% CI 2.1–6.8; FDR <0.001). Elevated odds were also observed for refuse and recycling workers, referring to waste collection and recycling occupations (OR 3.3, 95% CI 2.4–4.5; FDR <0.001), machine operators in food and textile production (OR 3.3, 95% CI 2.0–5.5; FDR <0.001), cleaning and facility staff (OR 3.2, 95% CI 2.3–4.6; FDR <0.001), food preparation workers (OR 3.2, 95% CI 2.2–4.5; FDR <0.001), and selected care and social service roles, including social workers and counselors (OR 2.5, 95% CI 1.7–3.7; FDR <0.001). Full occupation‐level estimates are shown in Table S5 and for the highest 40 OR in Fig. 2.

**Fig. 2:**
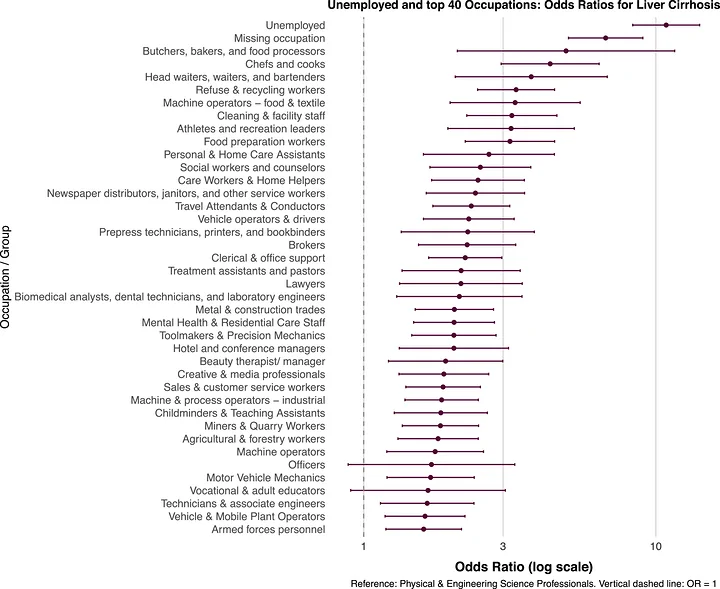
Odds ratios for incident cirrhosis by unemployment and occupational group, Sweden 2002–2021. Estimates from risk‐set–matched conditional logistic regression; reference = Physical & Engineering Science Professionals (SSYK 2012). Points show ORs with 95% confidence intervals (CIs); vertical dashed line mark OR = 1. “Missing occupation” denotes employed individuals without a recorded SSYK code in labor market studies (LISA).

For descriptive visualization and ease of interpretation, occupations were further aggregated into broader industry domains informed by the NACE classification, and crude occupation‐level estimates were displayed within each domain (Fig. S3a–c) [19]. Adjustment for education and household income modestly attenuated the effect estimates, but the overall pattern was preserved. Additional adjustment for pre‐index job‐change rate and unemployment proportion did not alter the rank ordering or direction of associations (Table S6). In addition, patterns were broadly similar across sex and age strata, although effect sizes were generally larger in men. Detailed subgroup estimates are presented in Tables S8 and S9.

### Etiology‐specific analyses

Associations varied by underlying etiology, although some patterns were shared. Unemployment was associated with the highest odds of alcohol‐related and viral hepatitis–related cirrhosis, and elevated odds were also observed across food‐service occupations, refuse and recycling work, and selected care and social service occupations. For MASLD, associations were less pronounced and were concentrated in a smaller set of occupations, including cleaning and facility staff and personal and home‐care roles. We did not observe significant associations for autoimmune‐related cirrhosis. Detailed etiologic estimates are shown in Fig. 3, Table S7a–c, and Fig. S4a–c.

**Fig. 3:**
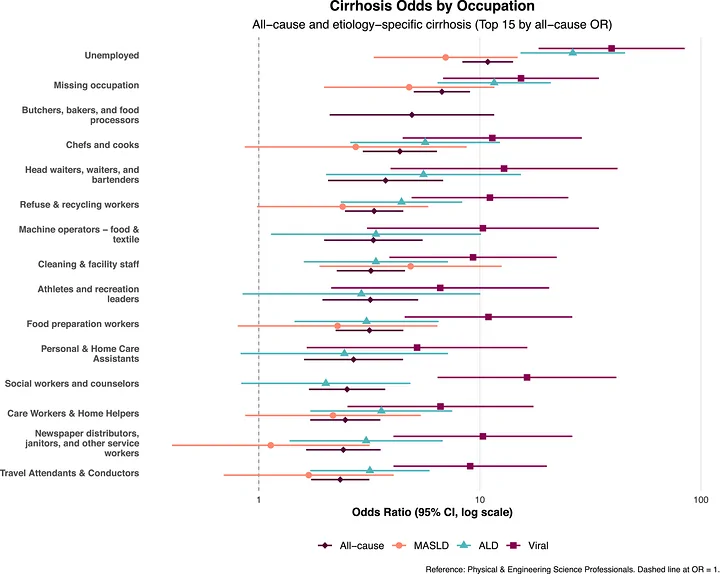
Etiology‐specific relative odds of incident cirrhosis by occupation, Sweden 2002–2021. Results from crude conditional logistic regression in the case–control cohort. Occupations (SSYK‐2012, three‐digit codes) are ordered and grouped by industry domain. Colored symbols show odds ratios (ORs) with 95% confidence intervals for viral, alcohol‐related, metabolic dysfunction–associated steatotic liver disease (MASLD)‐related, and all‐cause cirrhosis on a logarithmic scale; the dashed vertical line marks OR = 1, and the reference occupation is physical and engineering science professionals. CI, confidence interval.

### Sensitivity analyses

In a sensitivity analysis using occupation recorded 5 years before the index date, effect estimates were attenuated overall, particularly for unemployment, but the pattern of higher risk occupational groups remained similar. The OR for unemployment decreased from 10.9 to 6.7, whereas increased estimates for occupations such as food preparation and refuse and recycling work remained evident (Table S5).

## Discussion

In this nationwide case–control study, we found marked differences in incident cirrhosis by labor‐market status and occupation in Sweden. Unemployment was more common among cases than controls and was associated with the highest odds of cirrhosis. Among employed individuals, elevated odds were concentrated in selected occupational groups, particularly food preparation workers, refuse and recycling workers, and care and social service occupations. These patterns persisted after adjustment for socioeconomic indicators and remained evident, although attenuated, when occupation was assessed 5 years before diagnosis.

Individuals who were diagnosed with cirrhosis were more likely to drop out of the labor market early on, which is supported by higher proportion of individuals who obtained sick leave, disability benefits, and early retirement compared to controls. The attenuation in the 5‐year analysis is consistent with the possibility that labor‐market exit may partly reflect declining health before diagnosis, although reverse causation cannot be excluded. At the same time, unemployment was still the single strongest marker even when assessed further from diagnosis, suggesting that the finding is not explained solely by late‐stage disease causing labor‐market exit. From a clinical perspective, unemployment may therefore represent a pragmatic signal of increased likelihood of unrecognized advanced liver disease in working‐age adults, even if the pathways underlying that association are multifactorial rather than directly causal.

The employed individuals showed concentrated higher odds in certain occupations. Food preparation work, refuse and recycling, and selected care and social service roles are not traditionally highlighted in hepatology risk‐stratification frameworks, yet in this study they consistently emerged as occupations in which cirrhosis odds were the highest. Individuals with a missing occupational code may represent a heterogeneous group, potentially including people with weaker labor‐market attachment, including low‐intensity employment or administrative non‐capture [16]. For clinicians, this suggests that occupation may provide useful additional context when assessing whether a working‐age patient should undergo earlier liver evaluation.

The 5‐year sensitivity analysis supports the robustness of the occupational findings. Although estimates were attenuated overall, the pattern of higher risk occupations remained broadly similar, including for food preparation and refuse and recycling work. In contrast, the association with unemployment attenuated more substantially, suggesting that occupational group may capture a more stable dimension of long‐term social and work‐related vulnerability, whereas labor‐market status close to diagnosis may be more sensitive to short‐term health‐related changes.

The lower proportion of women in our cohort reflects the underlying sex distribution of cirrhosis in the source population, as cirrhosis is more common among men [2, 21]. As controls were matched to cases on sex, the higher proportion of men is unlikely to reflect selection bias but rather the real‐world epidemiology of the disease population. However, the smaller number of female cases reduces precision in sex‐stratified analyses and may limit the ability to detect occupation‐specific associations among women.

The etiologic analyses showed both shared and distinct patterns. Unemployment remained a strong marker across major etiologies, whereas among employed individuals, higher odds were concentrated in a smaller set of occupational groups. Some of these patterns may reflect differing constellations of social, behavioral, and work‐related risk factors across occupations. For example, viral hepatitis‐related cirrhosis was more common in occupations involving high interpersonal contact and social disadvantage, whereas alcohol‐related cirrhosis was more prominent in parts of the food and other service sectors. MASLD‐related cirrhosis appeared to be concentrated in a narrower range of lower income occupational groups. The etiology‐specific findings should be interpreted in the context of register‐based classification. The distribution of etiologies in our cohort differed from previous Nordic cirrhosis studies using medical‐record review, histopathology, or prospective case ascertainment with a higher reported proportion for alcohol‐ and MASLD‐related cirrhosis [22, 23, 24].

Our findings are contistent with previous studies showing that CLD and cirrhosis are more common among socioeconomically disadvantaged groups, and that less favorable socioeconomic circumstances are associated with worse outcomes [2, 21]. Similar gradients have been reported for specific liver diseases, including alcohol‐related liver disease and MASLD [25, 26]. In occupationally focused literature, a recent systematic review has suggested that shift work may be associated with increased MASLD risk [11]. In‐line with this, we observed elevated odds of cirrhosis in several occupations commonly characterized by shift work, night work, or otherwise irregular schedules, including care and residential support roles, travel‐related occupations, and food‐service work. In addition, our finding of higher odds of viral hepatitis‐related cirrhosis among refuse and recycling workers is supported by occupational studies reporting higher hepatitis B virus and hepatitis C virus prevalence among waste handlers, suggesting that waste‐related work environments may involve exposure pathways relevant to viral hepatitis risk [27]. However, our findings also extend the existing literature by showing that cirrhosis risk is not only socially patterned at a broad socioeconomic level but also concentrated in specific occupational groups among working‐age adults overall and by specific etiologies.

From a clinical perspective, these findings suggest that labor‐market and occupational information may help identify groups in whom cirrhosis is more likely to remain unrecognized until later stages of the disease. This interpretation is in line with recent liver‐specific literature showing that earlier detection increasingly depends on practical approaches to identifying at‐risk groups in addition to traditional hepatology settings. Community‐based pathways, targeted noninvasive testing strategies, and risk‐factor‐based entry criteria all emphasize that scalable case‐finding depends first on defining the right population [9, 28, 29].

Integrating occupational and labor‐market information into liver disease risk‐stratification frameworks might help reduce diagnostic delays and address social gradients in liver outcomes. Potential platforms for future evaluation include workplace, employer‐supported, and union‐linked health initiatives tailored to sectors with concentrated cirrhosis risk. Because labor‐market information is routinely collected in many European statistical systems, this approach may have relevance beyond Sweden, where linkage to health data is possible [19].

### Strengths and limitations

The study has several strengths. First, it was based on nationwide Swedish registry data spanning over two decades. Second, Sweden's universal healthcare system aims to provide equal and universal access to all levels of healthcare, which reduces selection bias due to labor‐market positioning [30]. Third, we performed a pre‐defined harmonization of occupational codes across changes in the Swedish classification system, which allowed consistent occupation‐based analyses over time. Fourth, in terms of generalizability, the incidence of cirrhosis in Sweden is comparable to that in other European countries, and classification of industries according to the European NACE system facilitates transferability of our findings to other European settings [1, 19].

Several limitations should also be acknowledged. First, the case–control design does not allow estimation of absolute risks, as the DELIVER cohort is constructed for relative risk assessment using matched sampling. Second, outcome and etiologic classification relied on register coding and may be affected by incomplete capture or misclassification, particularly for alcohol‐ and MASLD‐related cirrhosis, although the Swedish hospital registers have a high coverage and cirrhosis diagnoses have shown good validity in prior validation studies [25, 31]. Third, individual‐level data on alcohol consumption, body mass index, smoking, glycemic control, and other lifestyle‐related factors were unavailable, and residual confounding is therefore possible. Fourth, our main occupation analyses were restricted to individuals classified as employed or unemployed, so they do not capture the full labor‐market spectrum. Finally, reverse causation cannot be excluded, because labor‐market status and occupation were assessed close to diagnosis; although the 5‐year sensitivity analysis attenuated the estimates, particularly for unemployment, elevated odds remained for several occupational groups.

## Conclusion

Liver cirrhosis in Sweden was strongly patterned by labor‐market position and occupation, with marked concentration among unemployed individuals and several specific occupational groups. These findings suggest that routinely collected employment information may help identify working‐age adults who could benefit from earlier liver fibrosis assessment alongside established metabolic, alcohol‐related, and viral risk factors.

## Author contributions

**Marie Zalesiak**: Conceptualization; writing—original draft; methodology; visualization; writing—review and editing; software; formal analysis; project administration; investigation. **Juan Vaz**: Writing—review and editing. **Eralda Asllanaj**: Writing—review and editing. **Martin Henriksson**: Writing—review and editing. **Hannes Hagström**: Conceptualization; funding acquisition; supervision; resources; data curation; writing—review and editing. **Ying Shang**: Conceptualization; funding acquisition; resources; supervision; writing—review and editing; validation; investigation.

## Conflicts of interest statement

Ying Shang is supported by grants from the Swedish Research Council, the Swedish Cancer Society, Region Stockholm (CIMED and clinical researcher award), the Swedish Heart and Lung Foundation, and others. Hannes Hagström served as a consultant, speaker, or advisory board member for AstraZeneca, Boehringer Ingelheim, Bristol Myers Squibb, GSK, Echosens, Ipsen, MSD, and Novo Nordisk and served on hepatic event adjudication committees for Arrowhead, Boehringer Ingelheim, KOWA, and GW Pharma. Juan Vaz has received consulting fees from Eisai, AstraZeneca, and Madrigal Pharmaceuticals. Martin Henriksson has no competing interests to declare that are relevant to the content of this article. Marie Zalesiak is employed part‐time by Boehringer Ingelheim. Eralda Asllanaj is an employee of Boehringer Ingelheim. The manuscript has been handled by an external editor: Stefan Lindgren, Emeritus professor, Department of Clinical Sciences Malmö, Lund University, SE‐221 00 Lund, Sweden.

## Ethics statement

The study was approved by the Swedish Ethical Review Authority (Dnr. 2017/1019‐31/1). The study was based on pseudonymized data; informed consent was waived.

## Supporting information




**Table S1**. ICD‐10 code definitions and hierarchical classification algorithm used to define the primary outcome (incident liver cirrhosis) and etiology‐specific cirrhosis subgroups in Swedish national registers. Incident cirrhosis was defined as the first‐ever hospital discharge, specialist outpatient visit, or underlying/contributing cause of death fulfilling any of the codes listed for compensated or decompensated cirrhosis, including strongly indicative decompensation events. Etiology of incident cirrhosis was then classified hierarchically (alcohol‐related, autoimmune, chronic viral hepatitis, MASLD/MASH, toxic, Wilson's disease, hemochromatosis) based on the first occurrence of the corresponding ICD‐10 codes.
**Table S2**. Matching SSYK—Stepwise matching algorithm for harmonizing occupational categories.
**Table S3**. Merged exposure groups—Mapping of detailed three‐digit occupational categories to merged exposure groups.
**Table S4**. Industry mapping—Mapping of merged occupational exposure groups into 13 broader industry groups.
**Table S5**. Primary model and time of exposure robustness.
**Table S6**. Results of primary models.
**Table S7a**. Etiology viral—Odds ratios (ORs) with 95% confidence intervals for viral hepatitis–related cirrhosis by occupational group, using physical and engineering science professionals as the reference category.
**Table S7b**. Odds ratios (ORs) with 95% confidence intervals for ALD–related cirrhosis by occupational group, using physical and engineering science professionals as the reference category.
**Table S7c**. ORs with 95% confidence intervals for MASLD–related cirrhosis by occupational group, using physical and engineering science professionals as the reference category.
**Table S8**. ORs with 95% confidence intervals for cirrhosis by occupational group, using physical and engineering science professionals as the reference category.
**Table S9**. ORs with 95% confidence intervals for cirrhosis by occupational group, using physical & engineering science professionals as the reference category.
**Figure S1**. Flowchart of the harmonization procedure used to map SSYK‐96 occupations to SSYK2012 three‐digit groups.
**Figure S2**. Pre‐index labor market status among cases and matched controls.
**Figure S3a–c**. Relative odds of incident cirrhosis by occupation, Sweden 2002–2021.
**Figure S4a–c**. Etiology‐specific relative odds of incident cirrhosis by occupation, Sweden 2002–2021.
