# Supplementary material for: Employment status and occupation are associated with liver cirrhosis: A nationwide Swedish case–control study
Source: J Intern Med. 2026 Oct 7;300(6):832–43. doi: 10.1111/joim.70166 (PMC13741810; doi:10.1111/joim.70166)
Supplement: Supplementary file 1 [file JOIM-300-832-s001.docx]

**Supplementary Material**

Table of Contents

[**Classifications of labor market status**	1](#_Toc225018864)

[Occupational Code Harmonization	2](#_Toc225018865)

[Tables	2](#_Toc225018866)

[Figures	15](#_Toc225018867)

### **Classifications of labor market status**

Occupational status for the observation period 2002 - 2021 was obtained from LISA for the calendar year preceding the index date. Occupational information in LISA has 95% completeness. Because LISA updates an individual´s main occupation (defined as the job with highest taxable salary) each November, index dates occurring in November–December were assigned occupational information from the same calendar year to minimize misclassification. For instance, if a patient was diagnosed with cirrhosis in June 2017, we used their occupational information from November 2016; if the diagnosis occurred in December 2017, the occupational information from November 2017 was used. LISA’s main occupation refers to the job with the highest taxable salary, if a person has multiple occupations during a year.

In the full sample we defined five mutually exclusive pre-index labor statuses: Employed, unemployed, on disability or activity benefit, long-term sick leave, and other out of labor force. Individuals classified as gainfully employed in November with a valid three-digit occupational code were categorized as “employed with occupation”. Those employed but lacking an occupational code were classified as “employed with missing occupational code”. Missing occupational codes may occur due to several reasons, such as delayed or absent earning reports (e.g., seasonal work, self-employment, or low-paid or temporary employment below annual salary thresholds).

The information about the occupational group of an employed individual is classified according to the Swedish Standard Classification of Occupations (SSYK), which is based on the International Standard Classification of Occupations 2008 (ISCO-08). In 2014, Statistics Sweden transformed the occupational group coding system of the register from SSYK-96 to SSYK-2012. The change in coding included, i.e., the introduction of new occupational groups and the abolition of older groups. To ensure a uniform, temporally consistent definition of exposure, occupational codes across SSYK coding systems were harmonized to SSYK-2012 at the 3-digit level using a pre-specified algorithm.

Starting from the harmonized 3-digit SSYK-2012 groups, we applied a pre-specified semantic aggregation to create analysis groups that preserve functional similarity while improving power (e.g. we combined “Recycling workers” and “Refuse Collectors & Recycling Workers” to "Refuse & Recycling workers"). The complete mapping from 3-digit SSYK-2012 to analysis groups is provided in Supplementary table S2.

To limit sparse-data bias and improve model stability, we combined any group contributing <10 cases or <30 total observations (cases + controls) into one category. This group contained 2,274 individuals. After applying this rule, the final exposure comprised 68 occupational groups. All occupational groups contained at least ~100–250 individuals, ensuring adequate data support for stable estimation. For generalizability we display the distribution of odds across industries, each occupational group was classified into a broader industrial domain, which was classified according to the European statistical classification of economic activities (NACE) implemented in Sweden as SNI 2007 (Table S4).

## **Occupational Code Harmonization**

In 2014, Statistics Sweden transformed the occupational group coding system of the register from SSYK-96 to SSYK-2012. The change in coding included, i.e., the introduction of new occupational groups and the abolition of older groups. To ensure a uniform, temporally consistent definition of exposure, we mapped every observed SSYK-96 4-digit code (“undergrupp/ Fyrsifferniva”) (n = 355) in our dataset to the SSYK-2012 3-digit code (“yrkesgrupp/ Tresifferniva”), which comprises 147 groups.

For the mapping procedure we applied a pre-specified, 5-step hierarchical algorithm:

(i) exact 1:1 mapping from official translation key tables from SCB (117/355 ≈ 33.0%) (e.g. 2113 was defined as “chemist” in SSYK-96 and SSYK-2012).
(ii) (ii) pre-specified code translation based on the official SCB translation key (74/355 ≈ 20.8%) (e.g. 1231 was defined as “Finance managers and administrative managers” in SSYK-96 and was recommended to be translated to 121 “Chief Financial Officers” in SSYK-2012).
(iii) consistent prefix rule (100/ 355 ≈ 28.2%) (e.g. In SSYK-96 code, mechanical engineering technicians were coded as 3115, later mapping to SSYK-2012 codes as 3111 [engineers and technicians in industry, logistics, and production planning] or 3114 [engineers and technicians in mechanical engineering]. Since, our goal was to analyze the occupational groups on the broader 3-digit level, so we harmonized all these on the same SSYK 3-digit code 311 [Engineers and technicians]).
(iv) fuzzy text matching (19/ 355 ≈ 5.3%).

manual review by two investigators (45/ 355 ≈ 12.7%). This process yielded an exhaustive mapping (355/ 355; 100%) with zero codes remaining (see Supplementary Table S2 for the complete step log and Supplementary Figure S1 for the algorithm diagram).

## **Tables**

**Supplementary Table S1. ICD-10 code definitions and hierarchical classification algorithm used to define the primary outcome (incident liver cirrhosis) and etiology-specific cirrhosis subgroups in Swedish national registers.** Incident cirrhosis was defined as the first-ever hospital discharge, specialist outpatient visit, or underlying/contributing cause of death fulfilling any of the codes listed for compensated or decompensated cirrhosis, including strongly indicative decompensation events. Etiology of incident cirrhosis was then classified hierarchically (alcohol-related, autoimmune, chronic viral hepatitis, MASLD/MASH, toxic, Wilson’s disease, hemochromatosis) based on the first occurrence of the corresponding ICD-10 codes.

| **Outcome Category** | **Defining Criteria & ICD-10 Codes** |
| --- | --- |
| **Primary Outcome - Incident Liver Cirrhosis** | |
| **Any Incident cirrhosis** | First-ever register entry (primary and contributing diagnosis) for:   - Compensated cirrhosis   - Cirrhosis compensated: K74.6   - Esophageal varices (not bleeding): I85.9, I98.2   - Gastric varices (not bleeding): I86.4 - Decompensated cirrhosis   - Esophageal varices (bleeding): I85.0, I98.3   - Ascites: R18   - Hepatorenal syndrome: K76.7   - Liver failure: K72.1, K72.9   - Portal hypertension: K76.6 - Specific decompensation events strongly indicative of underlying cirrhosis - HBV-cirrhosis: B18.1E, B18.1G - HCV-cirrhosis: B18.2E, B18.2G - ALD-cirrhosis: K70.3 - Underlying or contributing cause of death recorded as any of the above |
| **ICD-10 Codes for Secondary Outcomes** | |
| **Etiology Classification of Incident Cirrhosis Cases**  **PMID: 32069337** | First-ever register entry (primary and contributing diagnosis) for:     - Alcohol-Related Liver Disease (ALD): K70.0, K70.3 (ALD-cirrhosis); or F10 (AUD) + K74.6 - Autoimmune liver disease (AIH, PBC, PSC): (K83.0A, K83.0F, K74.3, K75.4, (K50 or K51) + K83.0 - Chronic Viral Hepatitis:B18   - Chronic HCV: B18.2   - HCV-cirrhosis: B18.2E, B18.2G   - Chronic HBV: B18.1   - HBV-cirrhosis: B18.1E, B18.1G - MASLD & MASH: K76.0, K75.8, E66, E11 - Toxic liver disease: K71.- - Wilson’s Disease: E83.0B - Hemochromatosis: E83.1 |

**Supplementary Table S2. Matching SSYK - Stepwise matching algorithm for harmonizing occupational categories.** The table shows, for each rule applied in sequence (exact 1:1 matches, SCB key–based manual overrides, shared 3-digit prefixes, Swedish and English fuzzy‐matching rules, and final researcher overrides), the number of newly matched occupations (“n new”), cumulative number of matched occupations, remaining unmatched categories, and the percentage of all matches contributed by each step.

| **Step** | **n new** | **Cumulative** | **Remaining** | **% of total** |
| --- | --- | --- | --- | --- |
| Rule 1: exact 1:1 | 117 | 117 | 238 | 33.00% |
| Rule 2: manual overrides defined by SCB key | 74 | 191 | 164 | 20.80% |
| Rule 3: shared 3-digit prefix | 100 | 291 | 64 | 28.20% |
| Rule 4a: fuzzy SWE  (JW ≤ 0.25) | 15 | 306 | 49 | 4.20% |
| Rule 4b: fuzzy SWE (LV ≤ 1) | 0 | 306 | 49 | 0.00% |
| Rule 5a: fuzzy ENG  (JW ≤ 0.23) | 4 | 310 | 45 | 1.10% |
| Rule 5b: fuzzy ENG (LV ≤ 1) | 0 | 310 | 45 | 0.00% |
| Rule 6: manual overrides (2 researcher) | 45 | 355 | 0 | 12.70% |

**Supplementary Table S3. Merged Exposure Groups - Mapping of detailed 3-digit occupational categories to merged exposure groups.** The table shows how original Statistics Sweden occupational titles were collapsed into broader occupational groups used in the main analyses; categories not listed were retained unchanged (exposure_merged identical to the original code).

| **Merged exposure group** | **Original categories** |
| --- | --- |
| Armed forces personnel | Armed forces  Armed forces occupations  Armed forces personnel |
| Chief executives & senior managers | Chief executives and managing directors  Senior Officials & Managing Directors |
| Financial & administrative managers | Financial managers  Administrative and planning managers  Bank, finance, and insurance managers Purchasing, logistics, and transport managers |
| HR & personnel managers | Human resources managers  Personnel and HR managers |
| Sales & marketing managers | Sales and marketing managers  Retail managers  Business & Marketing Professionals |
| IT managers | IT managers |
| Research and development managers | Research and development managers |
| Social and welfare managers | Social and welfare managers |
| Other service managers | Other administrative and service managers  Other community service managers  Other service industry managers |
| Science & engineering professionals | Physicists and chemists  Mathematicians, actuaries, and statisticians  Civil engineers  Environmental and health protection specialists |
| Architects and surveyors | Architects and surveyors |
| Nurses | NursesNurses (continued)  Nurses (General) |
| Doctors | Doctors |
| Dentists | Dentists |
| Psychologists & psychotherapists | Psychologists and psychotherapists |
| Other healthcare specialists | Chiropractors, physiotherapists, and occupational therapists  Other healthcare specialists  Alternative medicine therapists |
| Veterinary professionals | Veterinarians  Veterinary nurses |
| Teaching professionals – University | University and higher education teachers  University & Higher Education Teachers |
| Teaching professionals – Secondary | High school teachers  Secondary School Teachers |
| Teaching professionals – Preschool/Primary | Primary school teachers, leisure-time pedagogues, and preschool teachers  Preschool managers  Primary School Teachers  Preschool Teachers & Aides |
| Vocational & adult educators | Vocational teachers  Other pedagogues with theoretical specialist competence |
| Business & financial professionals | Accountants & Statisticians  Auditors, financial analysts, and fund managers  Bank clerks and accountants  Insurance advisors, corporate salespeople, and buyers |
| HR & organization specialists | Organizational developers, investigators, and HR specialists |
| Marketing, PR & communication professionals | Marketers and communicators  PR & Communications Specialists  Information, communication, and PR managers |
| Lawyers | Lawyers |
| Law administration professionals | Legal secretaries, executive secretaries, and administrative assistants  Tax and social insurance administrators |
| Creative & media professionals | Authors, journalists, and interpreters  Artists, musicians, and actors Photographers, decorators, and entertainers  Designers and artists |
| Technicians & associate engineers | Engineers and technicians Operations, support, and network technicians  Audio, video, and lighting technicians  IT architects, system developers, and test leaders |
| Supervisors – construction & manufacturing | Supervisors in construction and manufacturing Operations managers in construction, civil engineering, and mining |
| Machine & process operators – industrial | Machine operators in chemical and pharmaceutical products  Process operators in the wood and paper industry  Other process and machine operators  Process and machine operators at steel and metal works  Machine operators in rubber, plastic, and paper industries  Paper & Pulp Plant Operators |
| Machine operators – food & textile | Machine operators in the food industry  Machine operators in textile, laundry, and leather industries |
| Electricians & repair technicians | Installation and industrial electricians  Electronics repairers and communication electricians |
| Vehicle operators & drivers | Car, motorcycle, and bicycle drivers  Truck and bus drivers  Train drivers and yard staff |
| Pilots & ship officers | Pilots, ship officers, and machine operators |
| Sailors & marine deck | Sailors and deckhands |
| Dock workers and ramp staff | Dock workers and ramp staff |
| Metal & construction trades | Foundry workers, welders, and sheet metal workers  Roofers, floor layers, and plumbers  Carpenters, bricklayers, and construction workers  Painters, varnishers, and chimney sweeps |
| Toolmakers & Precision Mechanics | Toolmakers & Precision Mechanics  Precision mechanics and craft workers |
| Wood & furniture trades | Surface finishers, woodworkers, and furniture makers |
| Textile & leather trades | Tailors, upholsterers, and leather workers |
| Chefs and cooks | Chefs and cold kitchen chefs |
| Kitchen managers & sous chefs | Head chefs and sous chefs  Head chefs, chefs and sous chefs |
| Food preparation workers | Fast food staff, kitchen and restaurant assistants  Food Preparation Assistants |
| Sales & customer service workers | Shop assistants  Cashiers  Travel agents, customer service staff, and receptionists  Event salespeople and telemarketers  Customer Service Staff  Retail Salespersons |
| Beauty therapist/ manager | Wellness, sports, and recreation managers  Beauty and body therapists |
| Travel Attendants & Conductors | Travel Attendants & Conductors  Cabin crew, train conductors, and guides |
| Clerical & office support | Office assistants and secretaries  HR & Payroll Administrators  Administrative & Executive Assistants  Office Clerks (Reference) |
| Education administrators | School principals and education administrators  Other education managers |
| Cleaning & facility staff | Cleaners and home service staff  Washers, window cleaners, and other cleaning workers  Cleaning supervisors and property caretakers |
| Refuse & recycling workers | Recycling workers  Refuse Collectors & Recycling Workers |
| Agricultural & forestry workers | Crop growers in agriculture and horticulture  Mixed crop and livestock farmers  Forestry workers  Animal breeders and caretakers  Fish farmers and fishermen  Berry pickers and planters |
| Police & security services | Police officers  Other security and surveillance occupations |
| Religious leaders & clergy | Priests and deacons  Religious leaders and managers |
| Other service personnel | Other service managers  Other service personnel |
| Unemployed | Unemployed |
| Missing occupation | Missing occupation |
| Newspaper distributors, janitors, and other service workers | Newspaper distributors, janitors, and other service workers |
| (unchanged categories) | Any exposure_group_2 value not listed above is carried over as-is (exposure_merged is identical to exposure_group_2). |

**Supplementary Table S4. Industry mapping - Mapping of merged occupational exposure groups into 13 broader industry groups.** The table lists, for each industry group, all included merged occupations (exposure_merged) used to construct industry-level categories in the main analyses; any occupations not listed were assigned to the “Other / Residual” category.

| **Industry group** | **Occupations (exposure_merged)** |
| --- | --- |
| **Healthcare & Social Care** | Nurses, Specialist Nurses, Nursing Assistants & Healthcare Aides, Doctors Dentists Psychologists & psychotherapists, Other healthcare specialists Biomedical analysts, dental technicians, and laboratory engineers Care Workers & Home Helpers Personal & Home Care Assistants Mental Health & Residential Care Staff Healthcare managers Elderly care managers Dental assistants Treatment assistants and pastors |
| **Education & Research** | Teaching professionals – University Teaching professionals – Secondary Teaching professionals – Preschool/Primary Vocational & adult educators Education administrators Childminders & Teaching Assistants Driving instructors and trainers Museum curators and librarians, Social workers and counselors |
| **Public Administration, Security & Defence** | Public Administrators & Analysts Police & security services Armed forces personnel Armed forces Armed forces occupations Politicians and senior officials Lawyers Law administration professionals, Religious leaders & clergy Social and welfare managers Elected representatives |
| **Business, Finance & Professional Services** | Chief executives & senior managers Financial & administrative managers HR & personnel managers HR & organization specialists Sales & marketing managers Business & financial professionals Marketing, PR & communication professionals Brokers Research and development managers IT managers Clerical & office support Creative & media professionals Science & engineering professionals Physical & Engineering Science Professionals Architects and surveyors Managers in architecture and engineering Technicians & associate engineers |
| **Manufacturing, Construction & Technical Trades** | Metal & construction trades Building & Construction Technicians Supervisors – construction & manufacturing Construction laborers Toolmakers & Precision Mechanics Wood & furniture trades Textile & leather trades Machine & process operators – industrial Machine operators – food & textile Machine operators Operations technicians and process supervisors Prepress technicians, printers, and bookbinders Electricians & repair technicians Motor Vehicle Mechanics Miners & Quarry Workers Ore processing workers and well drillers |
| **Transport & Mobile Work** | Vehicle operators & drivers Vehicle & Mobile Plant Operators Pilots & ship officers, Sailors & marine deck Travel Attendants & Conductors |
| **Logistics, Postal & Waste Services** | Warehouse staff and transport coordinators Mail carriers and postal workers Refuse & recycling workers Dock workers and ramp staff |
| **Hospitality, Food & Beverage** | Head waiters, waiters, and bartenders Head chefs and sous chefs Head chefs, chefs and sous chefs Kitchen managers & sous chefs, Chefs and cooks Food preparation workers Fast food staff, kitchen and restaurant assistants Butchers, bakers, and food processors Hotel, conferences & restaurant managers |
| **Retail & Customer-facing Services** | Retail Salespersons Sales & customer service worker s, Event salespeople and telemarketers Travel agents, customer service staff, and receptionists Shop assistants Cashiers Croupiers and collectors Athletes and recreation leaders Wellness, sports, and recreation managers Market and street vendors Beauty therapist/ manager |
| **Agriculture, Forestry & Environment** | Agricultural & forestry workers, Forestry and agricultural managers Biologists, pharmacologists, and specialists in agriculture and forestry |
| **Cleaning & Facility Support Services** | Cleaning & facility staff, Property and facility managers Newspaper distributors, janitors, and other service workers |
| **Not in Employment / Other** | Unemployed Missing occupation, Other residuals Other service personnel |
| **Other / Residual** | Any exposure_merged label not listed in the rows above (fallback category in the code). |

**Supplementary Table S5. Primary model and time of exposure robustness**

ORs with 95% confidence intervals for cirrhosis cases by occupational group and unemployed compared to the reference group Physical & Engineering Science Professionals. The table shows crude estimates, estimates from with an exposure defined 5 years prior to the index-date. BH denotes p-values adjusted for multiple testing using the Benjamini–Hochberg procedure.

| **Exposure** | **Primary OR (95% CI)** | **P BH** | **5y OR (95% CI)** | **5 y P BH** |
| --- | --- | --- | --- | --- |
| Unemployed | 10.94 (8.24–14.52) | <0.001 | 6.66 (4.76–9.32) | <0.001 |
| Missing occupation | 4.84 (3.45–6.79) | <0.001 | 3.54 (2.39–5.25) | <0.001 |
| Butchers, bakers, and food processors | 4.30 (1.66–11.17) | 0.007 |  |  |
| Chefs and cooks | 3.92 (2.59–5.95) | <0.001 | 3.86 (2.39–6.24) | <0.001 |
| Head waiters, waiters, and bartenders | 3.38 (1.77–6.45) | <0.001 | 3.87 (1.87–8.01) | 0.002 |
| Machine operators – food & textile | 3.24 (1.83–5.74) | <0.001 | 2.56 (1.36–4.82) | 0.012 |
| Food preparation workers | 2.99 (2.03–4.41) | <0.001 | 2.90 (1.83–4.61) | <0.001 |
| Occupation | 2.91 (2.06–4.10) | <0.001 | 2.62 (1.82–3.78) | <0.001 |
| Cleaning & facility staff | 2.81 (1.91–4.13) | <0.001 | 3.06 (1.87–5.01) | <0.001 |
| Personal & Home Care Assistants | 2.50 (1.48–4.22) | 0.002 |  |  |
| Athletes and recreation leaders | 2.45 (1.36–4.41) | 0.007 | 2.07 (1.06–4.04) | 0.077 |
| Newspaper distributors, janitors, and other service workers | 2.35 (1.55–3.57) | <0.001 | 1.55 (0.88–2.74) | 0.223 |
| Travel Attendants & Conductors | 2.32 (1.67–3.23) | <0.001 | 1.89 (1.30–2.75) | 0.004 |
| Care Workers & Home Helpers | 2.30 (1.58–3.36) | <0.001 | 1.72 (0.87–3.41) | 0.220 |
| Prepress technicians, printers, and bookbinders | 2.28 (1.26–4.13) | 0.015 | 2.81 (1.55–5.10) | 0.004 |
| Officers | 2.21 (1.04–4.72) | 0.066 | 1.69 (0.86–3.34) | 0.223 |
| Biomedical analysts, dental technicians, and laboratory engineers | 2.19 (1.26–3.80) | 0.014 | 1.53 (0.82–2.87) | 0.263 |
| Vehicle operators & drivers | 2.15 (1.46–3.15) | <0.001 | 2.13 (1.37–3.33) | 0.004 |
| Social workers and counselors | 2.09 (1.34–3.26) | 0.003 | 2.18 (1.36–3.48) | 0.004 |
| Treatment assistants and pastors | 2.06 (1.23–3.46) | 0.014 | 0.67 (0.33–1.36) | 0.349 |
| Mental Health & Residential Care Staff | 2.02 (1.43–2.84) | <0.001 | 1.75 (1.16–2.64) | 0.023 |
| Clerical & office support | 2.01 (1.47–2.74) | <0.001 | 1.92 (1.34–2.75) | 0.002 |
| Metal & construction trades | 1.97 (1.41–2.74) | <0.001 | 2.09 (1.45–3.02) | <0.001 |
| Hotel and conference managers | 1.90 (1.15–3.16) | 0.023 | 1.76 (1.08–2.88) | 0.063 |
| Toolmakers & Precision Mechanics | 1.90 (1.33–2.73) | 0.002 | 1.85 (1.25–2.75) | 0.008 |
| Beauty therapist/ manager | 1.90 (1.16–3.11) | 0.021 | 1.33 (0.78–2.24) | 0.375 |
| Childminders & Teaching Assistants | 1.89 (1.27–2.81) | 0.005 | 1.22 (0.74–2.01) | 0.522 |
| Sales & customer service workers | 1.85 (1.35–2.54) | <0.001 | 1.53 (1.07–2.20) | 0.055 |
| Brokers | 1.83 (1.19–2.82) | 0.014 | 2.27 (1.39–3.70) | 0.004 |
| Miners & Quarry Workers | 1.79 (1.30–2.48) | 0.002 | 1.86 (1.29–2.70) | 0.004 |
| Machine & process operators – industrial | 1.76 (1.28–2.41) | 0.002 | 1.85 (1.31–2.62) | 0.003 |
| Agricultural & forestry workers | 1.75 (1.24–2.47) | 0.004 | 1.48 (0.99–2.21) | 0.119 |
| Chief executives & senior managers | 1.74 (1.13–2.67) | 0.023 | 1.69 (1.07–2.68) | 0.063 |
| Machine operators | 1.73 (1.15–2.62) | 0.019 | 1.44 (0.89–2.31) | 0.225 |
| Nursing Assistants & Healthcare Aides | 1.71 (1.14–2.57) | 0.021 | 1.36 (0.87–2.15) | 0.263 |
| Technicians & associate engineers | 1.66 (1.12–2.46) | 0.022 | 1.77 (1.16–2.69) | 0.023 |
| Creative & media professionals | 1.64 (1.11–2.41) | 0.023 | 1.39 (0.91–2.15) | 0.223 |
| Vehicle & Mobile Plant Operators | 1.58 (1.12–2.22) | 0.019 | 1.54 (1.05–2.26) | 0.067 |
| Motor Vehicle Mechanics | 1.56 (1.07–2.28) | 0.035 | 1.34 (0.88–2.06) | 0.263 |
| Lawyers | 1.54 (0.88–2.69) | 0.195 | 1.09 (0.57–2.06) | 0.826 |
| Armed forces personnel | 1.48 (1.06–2.07) | 0.038 | 1.45 (1.03–2.05) | 0.080 |
| Business & financial professionals | 1.41 (0.98–2.02) | 0.103 | 1.29 (0.85–1.97) | 0.310 |
| Electricians & repair technicians | 1.40 (0.94–2.08) | 0.146 | 1.46 (0.96–2.21) | 0.157 |
| Operations technicians and process supervisors | 1.38 (0.84–2.26) | 0.282 | 1.60 (0.92–2.76) | 0.178 |
| Property and facility managers | 1.37 (0.77–2.41) | 0.370 | 1.11 (0.61–2.02) | 0.770 |
| Teaching professionals – Secondary | 1.35 (0.96–1.91) | 0.139 | 1.23 (0.83–1.82) | 0.384 |
| Specialist Nurses | 1.34 (0.97–1.85) | 0.120 | 1.39 (0.97–2.00) | 0.155 |
| Building & Construction Technicians | 1.28 (0.92–1.79) | 0.207 | 1.31 (0.91–1.89) | 0.228 |
| Nurses | 1.27 (0.85–1.91) | 0.340 | 1.11 (0.68–1.83) | 0.728 |
| Vocational & adult educators | 1.24 (0.62–2.49) | 0.652 |  |  |
| Teaching professionals – Preschool/Primary | 1.20 (0.83–1.74) | 0.415 | 1.08 (0.72–1.62) | 0.757 |
| Sales & marketing managers | 1.20 (0.83–1.73) | 0.415 | 1.30 (0.85–1.97) | 0.308 |
| Other (rare occupations) | 1.16 (0.83–1.60) | 0.475 | 1.15 (0.81–1.65) | 0.522 |
| Marketing, PR & communication professionals | 1.14 (0.73–1.80) | 0.660 | 1.42 (0.83–2.43) | 0.280 |
| Supervisors – construction & manufacturing | 1.12 (0.70–1.80) | 0.709 | 1.48 (0.83–2.61) | 0.263 |
| Teaching professionals – University | 1.12 (0.76–1.65) | 0.660 | 1.03 (0.67–1.60) | 0.878 |
| Mail carriers and postal workers | 1.09 (0.57–2.06) | 0.852 |  |  |
| Public Administrators & Analysts | 1.08 (0.76–1.53) | 0.730 | 1.13 (0.77–1.65) | 0.618 |
| Pilots & ship officers | 1.06 (0.60–1.90) | 0.862 | 1.54 (0.93–2.54) | 0.178 |
| Police & security services | 1.06 (0.66–1.71) | 0.856 | 1.23 (0.72–2.11) | 0.537 |
| Psychologists & psychotherapists | 1.03 (0.43–2.47) | 0.962 |  |  |
| Doctors | 1.00 (0.61–1.67) | 0.988 | 0.95 (0.54–1.66) | 0.870 |
| Physical & Engineering Science Professionals | 1.00 (NA–NA) |  | 1.00 (NA–NA) |  |
| Other healthcare specialists | 0.92 (0.50–1.71) | 0.852 | 1.20 (0.60–2.39) | 0.670 |
| Other service managers | 0.87 (0.54–1.41) | 0.660 | 1.24 (0.68–2.24) | 0.555 |
| Financial & administrative managers | 0.71 (0.39–1.29) | 0.347 |  |  |
| Dental assistants | 0.70 (0.27–1.76) | 0.539 |  |  |
| Natural science & statistics professionals | 0.61 (0.29–1.27) | 0.262 |  |  |

**Supplementary Table S6. Results primary models**

ORs with 95% confidence intervals for cirrhosis cases by occupational group and unemployed compared to the reference group Physical & Engineering Science Professionals. The table shows crude estimates, estimates from the within-SES model, and from the longitudinal occupational history model. P BH denotes p-values adjusted for multiple testing using the Benjamini–Hochberg procedure.

| **Exposure** | **OR Crude** | **P BH** | **OR Within-SES** | **P BH** | **OR longitudinal** | **P BH** |
| --- | --- | --- | --- | --- | --- | --- |
| Unemployed | 10.85 (8.34–14.13) | <0.001 | 7.76 (5.91–10.18) | <0.001 | 8.01 (6.06–10.57) | <0.001 |
| Missing occupation | 6.73 (5.02–9.03) | <0.001 | 5.32 (3.95–7.17) | <0.001 | 6.20 (4.62–8.32) | <0.001 |
| Butchers, bakers, and food processors | 4.92 (2.09–11.60) | <0.001 | 3.58 (1.52–8.47) | 0.012 | 4.79 (2.01–11.38) | 0.001 |
| Chefs and cooks | 4.34 (2.95–6.39) | <0.001 | 3.14 (2.12–4.65) | <0.001 | 4.11 (2.79–6.05) | <0.001 |
| Head waiters, waiters, and bartenders | 3.74 (2.05–6.81) | <0.001 | 2.68 (1.46–4.90) | 0.006 | 3.66 (2.01–6.67) | <0.001 |
| Refuse & recycling workers | 3.33 (2.46–4.51) | <0.001 | 2.51 (1.84–3.42) | <0.001 | 3.20 (2.36–4.34) | <0.001 |
| Machine operators – food & textile | 3.30 (1.98–5.51) | <0.001 | 2.53 (1.51–4.25) | 0.002 | 3.19 (1.91–5.32) | <0.001 |
| Cleaning & facility staff | 3.22 (2.26–4.60) | <0.001 | 2.38 (1.66–3.41) | <0.001 | 3.07 (2.15–4.38) | <0.001 |
| Athletes and recreation leaders | 3.19 (1.94–5.25) | <0.001 | 2.68 (1.63–4.42) | <0.001 | 3.08 (1.87–5.07) | <0.001 |
| Food preparation workers | 3.16 (2.23–4.50) | <0.001 | 2.29 (1.60–3.27) | <0.001 | 2.97 (2.08–4.23) | <0.001 |
| Personal & Home Care Assistants | 2.69 (1.60–4.51) | <0.001 | 2.07 (1.23–3.48) | 0.018 | 2.66 (1.58–4.47) | <0.001 |
| Social workers and counselors | 2.51 (1.69–3.73) | <0.001 | 2.34 (1.57–3.49) | <0.001 | 2.46 (1.65–3.67) | <0.001 |
| Care Workers & Home Helpers | 2.47 (1.71–3.55) | <0.001 | 1.94 (1.34–2.81) | 0.002 | 2.43 (1.69–3.50) | <0.001 |
| Newspaper distributors, janitors, and other service workers | 2.41 (1.64–3.56) | <0.001 | 1.76 (1.19–2.61) | 0.015 | 2.30 (1.56–3.39) | <0.001 |
| Travel Attendants & Conductors | 2.33 (1.72–3.16) | <0.001 | 1.73 (1.27–2.36) | 0.002 | 2.22 (1.63–3.00) | <0.001 |
| Religious leaders & clergy | 2.32 (1.31–4.13) | 0.007 | 2.24 (1.26–3.98) | 0.018 | 2.24 (1.26–3.97) | 0.011 |
| Vehicle operators & drivers | 2.29 (1.60–3.27) | <0.001 | 1.75 (1.22–2.51) | 0.010 | 2.16 (1.51–3.09) | <0.001 |
| Prepress technicians, printers, and bookbinders | 2.27 (1.34–3.84) | 0.005 | 1.68 (0.99–2.85) | 0.102 | 2.23 (1.32–3.77) | 0.006 |
| Brokers | 2.26 (1.54–3.32) | <0.001 | 1.97 (1.34–2.89) | 0.003 | 2.24 (1.53–3.29) | <0.001 |
| Clerical & office support | 2.23 (1.67–2.97) | <0.001 | 1.74 (1.30–2.33) | 0.002 | 2.20 (1.65–2.94) | <0.001 |
| Treatment assistants and pastors | 2.15 (1.35–3.43) | 0.003 | 2.11 (1.32–3.36) | 0.008 | 2.12 (1.33–3.38) | 0.004 |
| Lawyers | 2.15 (1.32–3.48) | 0.004 | 2.61 (1.60–4.27) | <0.001 | 2.08 (1.28–3.38) | 0.007 |
| Biomedical analysts, dental technicians, and laboratory engineers | 2.13 (1.30–3.49) | 0.005 | 1.96 (1.19–3.21) | 0.022 | 2.08 (1.27–3.41) | 0.008 |
| Metal & construction trades | 2.05 (1.50–2.79) | <0.001 | 1.56 (1.14–2.14) | 0.016 | 1.95 (1.43–2.65) | <0.001 |
| Mental Health & Residential Care Staff | 2.04 (1.48–2.80) | <0.001 | 1.63 (1.18–2.25) | 0.011 | 2.00 (1.45–2.75) | <0.001 |
| Toolmakers & Precision Mechanics | 2.04 (1.46–2.84) | <0.001 | 1.56 (1.11–2.19) | 0.025 | 1.98 (1.42–2.76) | <0.001 |
| Hotel and conference managers | 2.03 (1.32–3.13) | 0.003 | 1.69 (1.10–2.61) | 0.039 | 2.01 (1.30–3.09) | 0.004 |
| Beauty therapist/ manager | 1.90 (1.21–2.99) | 0.009 | 1.59 (1.01–2.50) | 0.091 | 1.87 (1.19–2.94) | 0.012 |
| Sales & customer service workers | 1.87 (1.39–2.51) | <0.001 | 1.45 (1.08–1.96) | 0.036 | 1.82 (1.36–2.45) | <0.001 |
| Machine & process operators – industrial | 1.85 (1.38–2.47) | <0.001 | 1.43 (1.06–1.92) | 0.039 | 1.82 (1.36–2.43) | <0.001 |
| Childminders & Teaching Assistants | 1.84 (1.27–2.65) | 0.003 | 1.40 (0.97–2.03) | 0.125 | 1.73 (1.20–2.50) | 0.007 |
| Miners & Quarry Workers | 1.83 (1.36–2.47) | <0.001 | 1.37 (1.01–1.86) | 0.086 | 1.74 (1.29–2.35) | <0.001 |
| Creative & media professionals | 1.81 (1.26–2.61) | 0.003 | 1.61 (1.12–2.33) | 0.028 | 1.76 (1.22–2.54) | 0.006 |
| Agricultural & forestry workers | 1.80 (1.31–2.47) | <0.001 | 1.36 (0.99–1.88) | 0.110 | 1.71 (1.25–2.35) | 0.002 |
| Machine operators | 1.76 (1.20–2.57) | 0.007 | 1.34 (0.91–1.97) | 0.198 | 1.71 (1.17–2.50) | 0.011 |
| Officers | 1.70 (0.88–3.29) | 0.153 | 1.84 (0.95–3.56) | 0.122 | 1.64 (0.85–3.17) | 0.187 |
| Motor Vehicle Mechanics | 1.69 (1.20–2.39) | 0.005 | 1.30 (0.92–1.85) | 0.198 | 1.65 (1.17–2.33) | 0.009 |
| Vocational & adult educators | 1.66 (0.90–3.05) | 0.147 | 1.67 (0.91–3.07) | 0.158 | 1.65 (0.90–3.05) | 0.147 |
| Technicians & associate engineers | 1.65 (1.14–2.38) | 0.013 | 1.40 (0.96–2.03) | 0.127 | 1.63 (1.13–2.36) | 0.016 |
| Vehicle & Mobile Plant Operators | 1.62 (1.18–2.22) | 0.005 | 1.22 (0.89–1.68) | 0.291 | 1.54 (1.12–2.11) | 0.013 |
| Armed forces personnel | 1.60 (1.19–2.16) | 0.004 | 1.44 (1.07–1.95) | 0.039 | 1.60 (1.19–2.16) | 0.005 |
| Chief executives & senior managers | 1.58 (1.07–2.33) | 0.035 | 1.83 (1.23–2.72) | 0.011 | 1.59 (1.08–2.35) | 0.033 |

**Supplementary Table S7A. Etiology Viral - Odds ratios (ORs) with 95% confidence intervals for viral hepatitis–related cirrhosis by occupational group, using Physical & Engineering Science Professionals as the reference category.** Estimates are derived from the primary conditional logistic regression model restricted to cirrhosis cases with underlying viral hepatitis etiology. P-values and P BH values are shown, the latter adjusted for multiple testing using the Benjamini–Hochberg procedure.

| **Table S7A - Viral Hepatitis** |  |  |  |
| --- | --- | --- | --- |
| **Exposure** | **OR (95% CI)** | **p-value** | **P-value (BH)** |
| Physical & Engineering Science Professionals | 1.00 (ref) | — | — |
| Unemployed | 36.62 (16.95–79.11) | < 0.001 | <0.01 |
| Missing occupation | 14.42 (6.35–32.79) | < 0.001 | <0.01 |
| Social workers and counselors | 14.39 (5.61–36.88) | < 0.001 | <0.01 |
| Head waiters, waiters, and bartenders | 11.93 (3.64–39.06) | < 0.001 | <0.01 |
| Newspaper distributors, janitors, and other service workers | 10.79 (4.20–27.68) | < 0.001 | <0.01 |
| Chefs and cooks | 10.62 (4.15–27.19) | < 0.001 | <0.01 |
| Food preparation workers | 10.30 (4.26–24.87) | < 0.001 | <0.01 |
| Refuse & recycling workers | 10.26 (4.49–23.44) | < 0.001 | <0.01 |
| Psychologists & psychotherapists | 9.53 (2.76–32.84) | < 0.001 | <0.01 |
| Travel Attendants & Conductors | 8.45 (3.76–18.97) | < 0.001 | <0.01 |
| Vehicle operators & drivers | 8.35 (3.43–20.33) | < 0.001 | <0.01 |
| Cleaning & facility staff | 8.19 (3.39–19.77) | < 0.001 | <0.01 |
| Toolmakers & Precision Mechanics | 7.41 (3.11–17.66) | < 0.001 | <0.01 |
| Mental Health & Residential Care Staff | 7.20 (3.08–16.79) | < 0.001 | <0.01 |
| Athletes and recreation leaders | 6.07 (1.93–19.05) | 0.00200 | <0.01 |
| Care Workers & Home Helpers | 6.05 (2.26–16.17) | < 0.001 | <0.01 |
| Treatment assistants and pastors | 5.64 (1.84–17.31) | 0.00248 | <0.01 |
| Personal & Home Care Assistants | 5.21 (1.63–16.67) | 0.00539 | 0.011 |
| Agricultural & forestry workers | 5.17 (2.19–12.19) | < 0.001 | <0.01 |
| Metal & construction trades | 5.15 (2.28–11.63) | < 0.001 | <0.01 |
| Miners & Quarry Workers | 4.87 (2.17–10.90) | < 0.001 | <0.01 |
| Childminders & Teaching Assistants | 4.37 (1.69–11.29) | 0.00235 | <0.01 |
| Machine operators | 3.91 (1.44–10.64) | 0.00765 | 0.016 |
| Vehicle & Mobile Plant Operators | 3.80 (1.66–8.68) | 0.00155 | <0.01 |
| Machine & process operators – industrial | 3.63 (1.61–8.17) | 0.00186 | <0.01 |
| Clerical & office support | 3.31 (1.45–7.54) | 0.00441 | <0.01 |
| Beauty therapist/ manager | 3.14 (0.94–10.46) | 0.06218 | 0.092 |
| Religious leaders & clergy | 3.13 (0.94–10.41) | 0.06232 | 0.092 |
| Technicians & associate engineers | 2.99 (1.19–7.52) | 0.01964 | 0.035 |
| Nursing Assistants & Healthcare Aides | 2.95 (1.10–7.90) | 0.03131 | 0.054 |
| Armed forces personnel | 2.95 (1.29–6.71) | 0.01013 | 0.020 |
| Sales & customer service workers | 2.94 (1.28–6.73) | 0.01088 | 0.021 |
| Prepress technicians, printers, and bookbinders | 2.89 (0.88–9.44) | 0.07953 | 0.114 |
| Electricians & repair technicians | 2.86 (1.09–7.49) | 0.03283 | 0.054 |
| Mail carriers and postal workers | 2.83 (0.87–9.28) | 0.08506 | 0.119 |
| Teaching professionals – Secondary | 2.81 (1.18–6.65) | 0.01918 | 0.035 |
| Brokers | 2.78 (1.01–7.63) | 0.04736 | 0.074 |
| Teaching professionals – Preschool/Primary | 2.65 (1.07–6.56) | 0.03479 | 0.056 |
| Supervisors – construction & manufacturing | 2.57 (0.87–7.62) | 0.08873 | 0.121 |
| Hotel and conference managers | 2.53 (0.79–8.10) | 0.11790 | 0.152 |
| Chief executives & senior managers | 2.39 (0.86–6.67) | 0.09579 | 0.127 |
| Police & security services | 2.27 (0.80–6.43) | 0.12258 | 0.155 |
| Creative & media professionals | 2.00 (0.75–5.34) | 0.16451 | 0.203 |
| Other (rare occupations) | 1.79 (0.76–4.22) | 0.18593 | 0.224 |
| Motor Vehicle Mechanics | 1.76 (0.66–4.74) | 0.26021 | 0.306 |
| Pilots & ship officers | 1.74 (0.54–5.62) | 0.35516 | 0.409 |
| Doctors | 1.46 (0.44–4.87) | 0.54104 | 0.585 |
| Business & financial professionals | 1.38 (0.54–3.54) | 0.49821 | 0.562 |
| Teaching professionals – University | 1.37 (0.49–3.83) | 0.55443 | 0.588 |
| Building & Construction Technicians | 1.34 (0.54–3.33) | 0.53497 | 0.585 |
| Specialist Nurses | 1.11 (0.46–2.71) | 0.81804 | 0.834 |
| Public Administrators & Analysts | 1.10 (0.43–2.81) | 0.83629 | 0.836 |
| Sales & marketing managers | 0.84 (0.27–2.55) | 0.75344 | 0.783 |

**Supplementary Table S7B. Odds ratios (ORs) with 95% confidence intervals for ALD–related cirrhosis by occupational group, using Physical & Engineering Science Professionals as the reference category.** Estimates are derived from the primary conditional logistic regression model restricted to cirrhosis cases with underlying ALD etiology. P-values and P BH values are shown, the latter adjusted for multiple testing using the Benjamini–Hochberg procedure.

| **Table S7B - ALD** |  |  |  |
| --- | --- | --- | --- |
| **Exposure** | **OR (95% CI)** | **p-value** | **P-value (BH)** |
| Physical & Engineering Science Professionals | 1.00 (ref) | — | — |
| Unemployed | 26.56 (15.37–45.87) | < 0.001 | <0.01 |
| Missing occupation | 11.81 (6.53–21.34) | < 0.001 | <0.01 |
| Chefs and cooks | 5.60 (2.56–12.25) | < 0.001 | <0.01 |
| Lawyers | 5.59 (2.46–12.70) | < 0.001 | <0.01 |
| Head waiters, waiters, and bartenders | 5.44 (1.96–15.07) | 0.00114 | <0.01 |
| Religious leaders & clergy | 4.58 (1.39–15.14) | 0.01258 | 0.025 |
| Refuse & recycling workers | 4.58 (2.43–8.65) | < 0.001 | <0.01 |
| Prepress technicians, printers, and bookbinders | 4.35 (1.58–11.97) | 0.00443 | 0.013 |
| Creative & media professionals | 3.67 (1.83–7.36) | < 0.001 | <0.01 |
| Specialist Nurses | 3.59 (1.99–6.46) | < 0.001 | <0.01 |
| Chief executives & senior managers | 3.57 (1.76–7.25) | < 0.001 | <0.01 |
| Operations technicians and process supervisors | 3.54 (1.48–8.47) | 0.00452 | 0.013 |
| Care Workers & Home Helpers | 3.50 (1.67–7.35) | < 0.001 | <0.01 |
| Machine operators – food & textile | 3.39 (1.13–10.14) | 0.02879 | 0.048 |
| Cleaning & facility staff | 3.28 (1.54–7.00) | 0.00208 | <0.01 |
| Biomedical analysts, dental technicians, and laboratory engineers | 3.25 (1.29–8.15) | 0.01227 | 0.025 |
| Clerical & office support | 3.25 (1.79–5.88) | < 0.001 | <0.01 |
| Metal & construction trades | 3.21 (1.73–5.93) | < 0.001 | <0.01 |
| Travel Attendants & Conductors | 3.17 (1.70–5.92) | < 0.001 | <0.01 |
| Newspaper distributors, janitors, and other service workers | 3.15 (1.42–7.03) | 0.00497 | 0.013 |
| Hotel and conference managers | 3.11 (1.34–7.22) | 0.00811 | 0.019 |
| Officers | 3.02 (0.96–9.52) | 0.05929 | 0.089 |
| Vehicle operators & drivers | 3.01 (1.47–6.20) | 0.00271 | 0.010 |
| Food preparation workers | 3.01 (1.42–6.39) | 0.00406 | 0.013 |
| Athletes and recreation leaders | 2.90 (0.84–10.01) | 0.09214 | 0.120 |
| Pilots & ship officers | 2.88 (1.31–6.36) | 0.00876 | 0.019 |
| Technicians & associate engineers | 2.86 (1.42–5.77) | 0.00338 | 0.012 |
| Brokers | 2.85 (1.26–6.43) | 0.01194 | 0.025 |
| Machine & process operators – industrial | 2.58 (1.42–4.68) | 0.00179 | <0.01 |
| Business & financial professionals | 2.57 (1.34–4.92) | 0.00460 | 0.013 |
| Motor Vehicle Mechanics | 2.56 (1.30–5.05) | 0.00655 | 0.016 |
| Sales & customer service workers | 2.50 (1.34–4.65) | 0.00400 | 0.013 |
| Mental Health & Residential Care Staff | 2.48 (1.27–4.83) | 0.00762 | 0.018 |
| Nurses | 2.47 (1.19–5.12) | 0.01530 | 0.029 |
| Marketing, PR & communication professionals | 2.45 (1.12–5.34) | 0.02425 | 0.042 |
| Personal & Home Care Assistants | 2.42 (0.82–7.13) | 0.10994 | 0.140 |
| Property and facility managers | 2.33 (0.93–5.86) | 0.07226 | 0.099 |
| Agricultural & forestry workers | 2.26 (1.19–4.30) | 0.01283 | 0.025 |
| Childminders & Teaching Assistants | 2.17 (0.98–4.80) | 0.05562 | 0.086 |
| Nursing Assistants & Healthcare Aides | 2.15 (0.98–4.73) | 0.05591 | 0.086 |
| Building & Construction Technicians | 2.06 (1.12–3.79) | 0.01956 | 0.036 |
| Miners & Quarry Workers | 2.06 (1.11–3.81) | 0.02172 | 0.038 |
| Other service managers | 2.05 (0.92–4.58) | 0.08040 | 0.107 |
| Machine operators | 2.05 (0.96–4.35) | 0.06300 | 0.092 |
| Electricians & repair technicians | 1.98 (0.94–4.15) | 0.07198 | 0.099 |
| Armed forces personnel | 1.93 (1.02–3.66) | 0.04294 | 0.070 |
| Social workers and counselors | 1.93 (0.80–4.66) | 0.14627 | 0.183 |
| Treatment assistants and pastors | 1.92 (0.71–5.23) | 0.19960 | 0.244 |
| Beauty therapist/ manager | 1.88 (0.70–5.04) | 0.21135 | 0.254 |
| Financial & administrative managers | 1.83 (0.60–5.53) | 0.28612 | 0.312 |
| Other (rare occupations) | 1.80 (0.96–3.36) | 0.06746 | 0.096 |
| Supervisors – construction & manufacturing | 1.63 (0.71–3.75) | 0.25356 | 0.287 |
| Teaching professionals – Secondary | 1.56 (0.77–3.14) | 0.21657 | 0.255 |
| Sales & marketing managers | 1.51 (0.75–3.07) | 0.25095 | 0.287 |
| Teaching professionals – University | 1.49 (0.72–3.05) | 0.28098 | 0.312 |
| Toolmakers & Precision Mechanics | 1.44 (0.68–3.07) | 0.34089 | 0.365 |
| Teaching professionals – Preschool/Primary | 1.27 (0.60–2.66) | 0.53065 | 0.549 |
| Public Administrators & Analysts | 1.26 (0.64–2.47) | 0.50501 | 0.532 |
| Vehicle & Mobile Plant Operators | 1.17 (0.58–2.35) | 0.66334 | 0.675 |
| Police & security services | 1.05 (0.42–2.62) | 0.92303 | 0.923 |

**Supplementary Table S7C. ORs with 95% confidence intervals for MASLD–related cirrhosis by occupational group, using Physical & Engineering Science Professionals as the reference category.** Estimates are derived from the primary conditional logistic regression model restricted to cirrhosis cases with underlying MASLD etiology. P-values and P BH values are shown, the latter adjusted for multiple testing using the Benjamini–Hochberg procedure.

| **Table7C. MASLD** |  |  |  |
| --- | --- | --- | --- |
| **Exposure** | **OR (95% CI)** | **p-value** | **P-value (BH)** |
| Physical & Engineering Science Professionals | 1.00 (ref) | — | — |
| Unemployed | 6.70 (3.11–14.46) | < 0.001 | <0.01 |
| Cleaning & facility staff | 4.48 (1.70–11.85) | 0.00249 | 0.037 |
| Missing occupation | 4.33 (1.75–10.74) | 0.00156 | 0.035 |
| Beauty therapist/ manager | 4.21 (1.29–13.75) | 0.01740 | 0.196 |
| Vehicle operators & drivers | 2.75 (1.01–7.52) | 0.04860 | 0.364 |
| Mental Health & Residential Care Staff | 2.73 (1.14–6.53) | 0.02434 | 0.219 |
| Operations technicians and process supervisors | 2.73 (0.82–9.07) | 0.10229 | 0.424 |
| Chefs and cooks | 2.53 (0.78–8.21) | 0.12251 | 0.424 |
| Refuse & recycling workers | 2.26 (0.91–5.59) | 0.07757 | 0.424 |
| Sales & customer service workers | 2.19 (0.95–5.05) | 0.06652 | 0.424 |
| Sales & marketing managers | 2.13 (0.84–5.40) | 0.11028 | 0.424 |
| Care Workers & Home Helpers | 2.12 (0.83–5.45) | 0.11858 | 0.424 |
| Toolmakers & Precision Mechanics | 2.10 (0.79–5.57) | 0.13520 | 0.435 |
| Clerical & office support | 2.03 (0.90–4.62) | 0.08922 | 0.424 |
| Vehicle & Mobile Plant Operators | 1.93 (0.78–4.78) | 0.15641 | 0.469 |
| Agricultural & forestry workers | 1.85 (0.77–4.46) | 0.17194 | 0.484 |
| Food preparation workers | 1.84 (0.64–5.34) | 0.26000 | 0.572 |
| Business & financial professionals | 1.83 (0.74–4.49) | 0.18892 | 0.500 |
| Supervisors – construction & manufacturing | 1.77 (0.59–5.27) | 0.30642 | 0.600 |
| Brokers | 1.75 (0.61–5.04) | 0.29650 | 0.600 |
| Machine & process operators – industrial | 1.74 (0.74–4.08) | 0.20333 | 0.508 |
| Teaching professionals – Secondary | 1.69 (0.68–4.21) | 0.26168 | 0.572 |
| Armed forces personnel | 1.65 (0.68–4.02) | 0.26715 | 0.572 |
| Childminders & Teaching Assistants | 1.59 (0.56–4.49) | 0.38088 | 0.705 |
| Technicians & associate engineers | 1.55 (0.53–4.56) | 0.42313 | 0.705 |
| Travel Attendants & Conductors | 1.47 (0.59–3.66) | 0.40761 | 0.705 |
| Metal & construction trades | 1.45 (0.59–3.57) | 0.41563 | 0.705 |
| Miners & Quarry Workers | 1.39 (0.56–3.43) | 0.47395 | 0.735 |
| Specialist Nurses | 1.38 (0.58–3.26) | 0.46420 | 0.735 |
| Electricians & repair technicians | 1.37 (0.50–3.75) | 0.53956 | 0.809 |
| Marketing, PR & communication professionals | 1.33 (0.40–4.47) | 0.64644 | 0.864 |
| Teaching professionals – Preschool/Primary | 1.29 (0.49–3.41) | 0.60744 | 0.864 |
| Creative & media professionals | 1.26 (0.42–3.78) | 0.68342 | 0.879 |
| Building & Construction Technicians | 1.23 (0.52–2.92) | 0.63345 | 0.864 |
| Machine operators | 1.14 (0.36–3.60) | 0.82832 | 0.979 |
| Nurses | 1.14 (0.37–3.52) | 0.82613 | 0.979 |
| Teaching professionals – University | 1.11 (0.40–3.11) | 0.83707 | 0.979 |
| Chief executives & senior managers | 1.06 (0.35–3.22) | 0.91715 | 0.987 |
| Other (rare occupations) | 1.05 (0.44–2.50) | 0.90439 | 0.987 |
| Public Administrators & Analysts | 1.04 (0.39–2.79) | 0.94319 | 0.987 |
| Newspaper distributors, janitors, and other service workers | 1.02 (0.36–2.89) | 0.97068 | 0.988 |
| Other service managers | 0.99 (0.29–3.40) | 0.98761 | 0.988 |
| Police & security services | 0.94 (0.25–3.52) | 0.93256 | 0.987 |
| Nursing Assistants & Healthcare Aides | 0.89 (0.26–2.99) | 0.84880 | 0.979 |
| Motor Vehicle Mechanics | 0.77 (0.25–2.39) | 0.65265 | 0.864 |

**Supplementary Table 8. ORs with 95% confidence intervals for cirrhosis by occupational group, using Physical & Engineering Science Professionals as the reference category.** Estimates are derived from the crude primary conditional logistic regression model stratified by sex. P BH values are shown, the latter adjusted for multiple testing using the Benjamini–Hochberg procedure.

| **Exposure** | **Men OR**  **(95% CI)** | **Women OR (95% CI)** | **Men (BH)** | **Women (BH)** |
| --- | --- | --- | --- | --- |
| Unemployed | 11.98 (9.00–15.94) | 7.03 (3.40–14.55) | <0.01 | <0.01 |
| Missing occupation | 7.43 (5.41–10.22) | 3.87 (1.76–8.52) | <0.01 | 0.019 |
| Butchers, bakers, and food processors | 6.85 (2.72–17.25) | NA | <0.01 | NA |
| Chefs and cooks | 5.38 (3.43–8.42) | 2.34 (0.97–5.65) | <0.01 | 0.369 |
| Food preparation workers | 4.40 (2.87–6.74) | 1.65 (0.74–3.67) | <0.01 | 0.559 |
| Childminders & Teaching Assistants | 4.09 (2.31–7.25) | 1.05 (0.48–2.28) | <0.01 | 0.960 |
| Cleaning & facility staff | 3.79 (2.50–5.76) | 2.07 (0.91–4.70) | <0.01 | 0.369 |
| Care Workers & Home Helpers | 3.60 (1.72–7.51) | 1.67 (0.78–3.57) | <0.01 | 0.515 |
| Machine operators – food & textile | 3.40 (1.91–6.07) | 2.55 (0.80–8.17) | <0.01 | 0.424 |
| Refuse & recycling workers | 3.40 (2.38–4.85) | 2.57 (1.21–5.45) | <0.01 | 0.136 |
| Athletes and recreation leaders | 3.39 (1.86–6.16) | 2.27 (0.82–6.29) | <0.01 | 0.424 |
| Religious leaders & clergy | 3.21 (1.69–6.09) | NA | <0.01 | NA |
| Head waiters, waiters, and bartenders | 3.00 (1.42–6.33) | 4.35 (1.36–13.91) | <0.01 | 0.136 |
| Personal & Home Care Assistants | 2.98 (1.25–7.12) | 1.95 (0.81–4.70) | 0.025 | 0.424 |
| Lawyers | 2.85 (1.68–4.86) | NA | <0.01 | NA |
| Social workers and counselors | 2.73 (1.58–4.72) | 1.89 (0.84–4.28) | <0.01 | 0.424 |
| Prepress technicians, printers, and bookbinders | 2.65 (1.53–4.57) | NA | <0.01 | NA |
| Newspaper distributors, janitors, and other service workers | 2.63 (1.73–4.00) | 1.49 (0.53–4.20) | <0.01 | 0.743 |
| Travel Attendants & Conductors | 2.50 (1.79–3.48) | 1.56 (0.70–3.45) | <0.01 | 0.640 |
| Vehicle operators & drivers | 2.28 (1.56–3.32) | 4.73 (1.32–16.88) | <0.01 | 0.136 |
| Brokers | 2.21 (1.44–3.40) | 2.30 (0.92–5.74) | <0.01 | 0.369 |
| Mental Health & Residential Care Staff | 2.19 (1.48–3.24) | 1.49 (0.70–3.16) | <0.01 | 0.655 |
| Metal & construction trades | 2.16 (1.56–2.99) | NA | <0.01 | NA |
| Biomedical analysts, dental technicians, and laboratory engineers | 2.14 (1.14–4.02) | 1.58 (0.61–4.12) | 0.030 | 0.655 |
| Hotel and conference managers | 2.12 (1.33–3.39) | 1.47 (0.48–4.51) | <0.01 | 0.759 |
| Toolmakers & Precision Mechanics | 2.08 (1.46–2.96) | 2.27 (0.77–6.72) | <0.01 | 0.424 |
| Clerical & office support | 1.97 (1.42–2.75) | 1.99 (0.95–4.16) | <0.01 | 0.369 |
| Creative & media professionals | 1.92 (1.27–2.90) | 1.31 (0.55–3.13) | <0.01 | 0.759 |
| Miners & Quarry Workers | 1.91 (1.39–2.61) | NA | <0.01 | NA |
| Sales & customer service workers | 1.90 (1.35–2.69) | 1.46 (0.70–3.05) | <0.01 | 0.655 |
| Vocational & adult educators | 1.88 (0.95–3.74) | NA | 0.103 | NA |
| Agricultural & forestry workers | 1.87 (1.33–2.62) | 1.42 (0.57–3.57) | <0.01 | 0.743 |
| Machine & process operators – industrial | 1.79 (1.30–2.47) | 1.68 (0.79–3.58) | <0.01 | 0.509 |
| Machine operators | 1.77 (1.19–2.64) | NA | <0.01 | NA |
| Motor Vehicle Mechanics | 1.76 (1.23–2.51) | NA | <0.01 | NA |
| Treatment assistants and pastors | 1.75 (1.02–3.00) | 3.76 (1.31–10.84) | 0.068 | 0.136 |
| Business & financial professionals | 1.69 (1.16–2.45) | 1.02 (0.46–2.28) | 0.012 | 0.960 |
| Vehicle & Mobile Plant Operators | 1.68 (1.21–2.34) | 1.88 (0.50–6.99) | <0.01 | 0.655 |
| Property and facility managers | 1.67 (1.01–2.78) | NA | 0.072 | NA |
| Technicians & associate engineers | 1.67 (1.13–2.47) | 1.55 (0.52–4.68) | 0.020 | 0.743 |
| Chief executives & senior managers | 1.67 (1.10–2.52) | NA | 0.026 | NA |
| Psychologists & psychotherapists | 1.66 (0.64–4.30) | 1.23 (0.38–3.97) | 0.345 | 0.855 |
| Officers | 1.66 (0.84–3.27) | NA | 0.188 | NA |
| Teaching professionals – Secondary | 1.65 (1.06–2.55) | 1.02 (0.49–2.14) | 0.042 | 0.960 |
| Electricians & repair technicians | 1.62 (1.11–2.36) | NA | 0.021 | NA |
| Specialist Nurses | 1.55 (1.13–2.14) | 1.28 (0.56–2.92) | 0.014 | 0.759 |
| Teaching professionals – Preschool/Primary | 1.53 (0.99–2.37) | 0.85 (0.40–1.82) | 0.083 | 0.826 |
| Marketing, PR & communication professionals | 1.51 (0.95–2.40) | NA | 0.109 | NA |
| Nursing Assistants & Healthcare Aides | 1.45 (0.89–2.37) | 1.28 (0.57–2.87) | 0.182 | 0.759 |
| Nurses | 1.41 (0.85–2.34) | 1.04 (0.47–2.30) | 0.213 | 0.960 |
| Operations technicians and process supervisors | 1.40 (0.87–2.27) | NA | 0.206 | NA |
| Building & Construction Technicians | 1.40 (1.01–1.94) | 0.59 (0.19–1.84) | 0.068 | 0.668 |
| Supervisors – construction & manufacturing | 1.39 (0.89–2.17) | NA | 0.188 | NA |
| Armed forces personnel | 1.39 (0.98–1.97) | 1.44 (0.68–3.02) | 0.100 | 0.655 |
| Sales & marketing managers | 1.32 (0.91–1.93) | 1.04 (0.45–2.40) | 0.188 | 0.960 |
| Mail carriers and postal workers | 1.30 (0.71–2.39) | NA | 0.432 | NA |
| Other (rare occupations) | 1.29 (0.91–1.82) | 0.87 (0.40–1.90) | 0.192 | 0.855 |
| Pilots & ship officers | 1.29 (0.78–2.11) | NA | 0.364 | NA |
| Police & security services | 1.22 (0.77–1.95) | 1.31 (0.39–4.44) | 0.432 | 0.826 |
| Beauty therapist/ manager | 1.22 (0.64–2.31) | 2.16 (0.90–5.15) | 0.576 | 0.369 |
| Public Administrators & Analysts | 1.21 (0.85–1.71) | 0.55 (0.20–1.53) | 0.336 | 0.611 |
| Teaching professionals – University | 1.16 (0.76–1.78) | 1.06 (0.47–2.36) | 0.530 | 0.960 |
| Other healthcare specialists | 1.02 (0.41–2.55) | 0.72 (0.28–1.83) | 0.982 | 0.759 |
| Physical & Engineering Science Professionals | 1.00 (ref) | 1.00 (ref) | — | — |
| Doctors | 0.99 (0.58–1.71) | 0.70 (0.25–2.02) | 0.984 | 0.759 |
| Other service managers | 0.90 (0.53–1.54) | 1.24 (0.48–3.16) | 0.733 | 0.826 |
| Dental assistants | NA | 0.78 (0.28–2.16) | NA | 0.826 |
| Science & engineering professionals | 0.71 (0.35–1.45) | NA | 0.386 | NA |
| Financial & administrative managers | 0.61 (0.30–1.24) | 1.03 (0.32–3.27) | 0.206 | 0.960 |

**Supplementary Table 9. ORs with 95% confidence intervals for cirrhosis by occupational group, using Physical & Engineering Science Professionals as the reference category.** Estimates are derived from the crude primary conditional logistic regression model stratified by age groups. P-values and P BH values are shown, the latter adjusted for multiple testing using the Benjamini–Hochberg procedure.

| Exposure | 35–49y OR (95% CI) | 50–65y OR (95% CI) | 35–49y (BH) | 50–65y (BH) |
| --- | --- | --- | --- | --- |
| Unemployed | 10.49 (6.54–16.81) | 10.93 (7.95–15.04) | <0.01 | <0.01 |
| Missing occupation | 6.01 (3.51–10.29) | 7.02 (4.94–9.97) | <0.01 | <0.01 |
| Butchers, bakers, and food processors | NA | 4.33 (1.48–12.63) | NA | 0.015 |
| Chefs and cooks | 5.38 (2.87–10.11) | 3.53 (2.14–5.82) | <0.01 | <0.01 |
| Machine operators – food & textile | 3.06 (1.33–7.03) | 3.40 (1.76–6.56) | 0.037 | <0.01 |
| Cleaning & facility staff | 2.75 (1.36–5.57) | 3.38 (2.23–5.12) | 0.024 | <0.01 |
| Athletes and recreation leaders | 3.45 (1.47–8.09) | 3.07 (1.65–5.70) | 0.023 | <0.01 |
| Head waiters, waiters, and bartenders | 4.21 (1.65–10.71) | 3.00 (1.34–6.71) | 0.021 | 0.015 |
| Refuse & recycling workers | 4.34 (2.52–7.47) | 3.00 (2.08–4.33) | <0.01 | <0.01 |
| Food preparation workers | 3.41 (1.85–6.30) | 3.00 (1.95–4.61) | <0.01 | <0.01 |
| Lawyers | NA | 2.74 (1.61–4.68) | NA | <0.01 |
| Care Workers & Home Helpers | 1.85 (0.87–3.94) | 2.72 (1.78–4.15) | 0.234 | <0.01 |
| Newspaper distributors, janitors, and other service workers | 1.53 (0.63–3.74) | 2.69 (1.73–4.18) | 0.568 | <0.01 |
| Social workers and counselors | 2.08 (0.95–4.58) | 2.67 (1.67–4.25) | 0.181 | <0.01 |
| Personal & Home Care Assistants | 3.10 (1.21–7.96) | 2.52 (1.36–4.67) | 0.069 | <0.01 |
| Clerical & office support | 1.62 (0.94–2.77) | 2.50 (1.77–3.52) | 0.193 | <0.01 |
| Hotel and conference managers | 1.07 (0.42–2.72) | 2.44 (1.49–4.01) | 0.928 | <0.01 |
| Biomedical analysts, dental technicians, and laboratory engineers | 1.47 (0.52–4.10) | 2.38 (1.35–4.21) | 0.652 | <0.01 |
| Vehicle operators & drivers | 2.43 (1.16–5.10) | 2.30 (1.52–3.47) | 0.069 | <0.01 |
| Metal & construction trades | 1.55 (0.87–2.77) | 2.22 (1.54–3.21) | 0.255 | <0.01 |
| Mental Health & Residential Care Staff | 1.77 (0.98–3.19) | 2.20 (1.51–3.22) | 0.179 | <0.01 |
| Travel Attendants & Conductors | 2.99 (1.69–5.31) | 2.19 (1.53–3.14) | <0.01 | <0.01 |
| Treatment assistants and pastors | 2.27 (0.94–5.48) | 2.17 (1.25–3.75) | 0.181 | 0.014 |
| Religious leaders & clergy | 2.82 (0.97–8.19) | 2.15 (1.09–4.26) | 0.179 | 0.045 |
| Brokers | 2.96 (1.41–6.20) | 2.15 (1.37–3.38) | 0.023 | <0.01 |
| Sales & customer service workers | 1.50 (0.87–2.57) | 2.03 (1.43–2.89) | 0.260 | <0.01 |
| Toolmakers & Precision Mechanics | 2.06 (1.13–3.77) | 2.02 (1.36–3.01) | 0.069 | <0.01 |
| Miners & Quarry Workers | 1.45 (0.83–2.55) | 2.00 (1.40–2.85) | 0.337 | <0.01 |
| Agricultural & forestry workers | 1.21 (0.62–2.38) | 1.97 (1.36–2.84) | 0.718 | <0.01 |
| Machine & process operators – industrial | 1.63 (0.96–2.76) | 1.94 (1.37–2.75) | 0.181 | <0.01 |
| Psychologists & psychotherapists | NA | 1.91 (0.86–4.24) | NA | 0.144 |
| Beauty therapist/ manager | 2.00 (0.88–4.57) | 1.89 (1.10–3.24) | 0.223 | 0.037 |
| Childminders & Teaching Assistants | 1.81 (0.94–3.49) | 1.87 (1.20–2.91) | 0.192 | 0.014 |
| Creative & media professionals | 1.71 (0.86–3.38) | 1.85 (1.20–2.85) | 0.242 | 0.014 |
| Machine operators | 1.58 (0.75–3.35) | 1.83 (1.17–2.86) | 0.389 | 0.015 |
| Prepress technicians, printers, and bookbinders | 4.07 (1.58–10.47) | 1.76 (0.92–3.36) | 0.023 | 0.120 |
| Property and facility managers | 0.92 (0.32–2.66) | 1.72 (0.99–3.01) | 0.928 | 0.081 |
| Operations technicians and process supervisors | NA | 1.71 (1.02–2.88) | NA | 0.064 |
| Business & financial professionals | 1.03 (0.53–2.02) | 1.71 (1.16–2.51) | 0.954 | 0.014 |
| Motor Vehicle Mechanics | 1.69 (0.92–3.12) | 1.68 (1.11–2.55) | 0.212 | 0.028 |
| Chief executives & senior managers | 1.33 (0.58–3.05) | 1.68 (1.07–2.63) | 0.667 | 0.042 |
| Nursing Assistants & Healthcare Aides | 1.24 (0.59–2.62) | 1.67 (1.08–2.60) | 0.718 | 0.039 |
| Officers | NA | 1.66 (0.79–3.49) | NA | 0.227 |
| Technicians & associate engineers | 1.66 (0.87–3.15) | 1.65 (1.05–2.58) | 0.242 | 0.048 |
| Electricians & repair technicians | 1.29 (0.65–2.59) | 1.64 (1.07–2.50) | 0.652 | 0.040 |
| Specialist Nurses | 1.23 (0.71–2.14) | 1.64 (1.14–2.34) | 0.652 | 0.015 |
| Vehicle & Mobile Plant Operators | 1.91 (1.04–3.50) | 1.57 (1.08–2.28) | 0.121 | 0.032 |
| Supervisors – construction & manufacturing | 0.84 (0.35–2.02) | 1.53 (0.93–2.53) | 0.798 | 0.129 |
| Sales & marketing managers | 0.87 (0.46–1.66) | 1.53 (1.02–2.29) | 0.781 | 0.059 |
| Teaching professionals – Secondary | 1.15 (0.64–2.06) | 1.53 (1.05–2.23) | 0.766 | 0.045 |
| Mail carriers and postal workers | NA | 1.48 (0.79–2.78) | NA | 0.268 |
| Building & Construction Technicians | 0.94 (0.52–1.69) | 1.45 (1.01–2.09) | 0.907 | 0.067 |
| Nurses | 1.36 (0.66–2.78) | 1.44 (0.92–2.23) | 0.629 | 0.141 |
| Vocational & adult educators | NA | 1.42 (0.71–2.84) | NA | 0.385 |
| Other (rare occupations) | 0.79 (0.43–1.46) | 1.40 (0.97–2.01) | 0.652 | 0.098 |
| Armed forces personnel | 2.20 (1.30–3.72) | 1.35 (0.94–1.95) | 0.023 | 0.141 |
| Teaching professionals – Preschool/Primary | 1.19 (0.63–2.23) | 1.31 (0.88–1.95) | 0.718 | 0.227 |
| Pilots & ship officers | 0.97 (0.34–2.78) | 1.30 (0.75–2.26) | 0.972 | 0.393 |
| Police & security services | 1.01 (0.43–2.37) | 1.28 (0.77–2.13) | 0.977 | 0.387 |
| Teaching professionals – University | 1.27 (0.66–2.44) | 1.22 (0.80–1.86) | 0.652 | 0.401 |
| Doctors | NA | 1.11 (0.65–1.90) | NA | 0.726 |
| Public Administrators & Analysts | 1.08 (0.62–1.90) | 1.10 (0.73–1.65) | 0.876 | 0.684 |
| Marketing, PR & communication professionals | 1.55 (0.74–3.24) | 1.10 (0.64–1.87) | 0.414 | 0.761 |
| Other healthcare specialists | 0.75 (0.27–2.12) | 1.03 (0.53–2.03) | 0.718 | 0.941 |
| Dental assistants | NA | 1.00 (0.41–2.45) | NA | 0.999 |
| Physical & Engineering Science Professionals | 1.00 (ref) | 1.00 (ref) | — | — |
| Other service managers | 1.84 (0.85–3.98) | 0.85 (0.49–1.47) | 0.242 | 0.608 |
| Financial & administrative managers | 0.71 (0.25–2.02) | 0.77 (0.38–1.56) | 0.681 | 0.509 |
| Science & engineering professionals | NA | 0.70 (0.34–1.44) | NA | 0.385 |

## **Figures**

**Supplementary Figure S1. Flowchart of the harmonization procedure used to map SSYK-96 occupations to SSYK2012 3-digit groups.** Starting from all SSYK-96 codes, we first assigned a 2012 code when an exact 1:1 crosswalk existed. Remaining codes were checked against a set of pre-specified expert overrides, then against SSYK2012 codes sharing a common 3-digit prefix. For codes still unmatched, we applied fuzzy string matching to Swedish job titles (Jaro–Winkler distance ≤ 0.25). Any residual unmapped codes were resolved by manual review, with two raters agreeing on the final 3-digit prefix.

**Supplementary Figure S2. Pre-index labor market status among cases and matched controls.** 100% stacked bars show the distribution of labor market categories during the year before the index date; labels indicate within-group percentages. Disability and Activity (early retirement) benefits are presented on one combined bar.

**Supplementary Figure S3 A.**

**Supplementary Figure S3 B.**

**Supplementary Figure S3 C.**

**Supplementary Figure S3A-C. Relative odds of incident cirrhosis by occupation, Sweden 2002–2021**. Results from crude conditional logistic regression. Occupations (SSYK-2012, 3-digit) are grouped by industry domain. Points show odds ratios with 95% CIs on a log scale; dashed line marks OR = 1 and the reference occupation is Physical & Engineering Science Professionals.

**Supplementary Figure S4 A.**

**Supplementary Figure S4 B.**

**Supplementary Figure S4 C**

**Supplementary Figure S4 A-C. Etiology-specific relative odds of incident cirrhosis by occupation, Sweden 2002–2021.** Results from crude conditional logistic regression in the case–control cohort. Occupations (SSYK-2012, three-digit codes) are ordered and grouped by industry domain. Colored symbols show ORs with 95% confidence intervals for viral (A), alcohol-related (B), and MASLD-related (C) on a logarithmic scale; the dashed vertical line marks OR = 1, and the reference occupation is Physical & Engineering Science Professionals.
